# A Combined RNA Signature Predicts Recurrence Risk of Stage I-IIIA Lung Squamous Cell Carcinoma

**DOI:** 10.3389/fgene.2021.676464

**Published:** 2021-06-14

**Authors:** Li Sun, Juan Li, Xiaomeng Li, Xuemei Yang, Shujun Zhang, Xue Wang, Nan Wang, Kanghong Xu, Xinquan Jiang, Yi Zhang

**Affiliations:** ^1^School of Public Health, Shandong First Medical University & Shandong Academy of Medical Sciences, Taian, China; ^2^Department of Clinical Laboratory, The Second Hospital, Cheeloo College of Medicine, Shandong University, Jinan, China; ^3^Department of Hematology, Jining First People’s Hospital, Jining, China; ^4^Respiratory and Critical Care Medicine Department, Qilu Hospital, Shandong University, Jinan, China

**Keywords:** lung squamous cell carcinoma, recurrence, TCGA, RNA signature, biomarker

## Abstract

**Objective:**

Recurrence remains the main cause of the poor prognosis in stage I-IIIA lung squamous cell carcinoma (LUSC) after surgical resection. In the present study, we aimed to identify the long non-coding RNAs (lncRNAs), microRNAs (miRNAs), and messenger RNAs (mRNAs) related to the recurrence of stage I-IIIA LUSC. Moreover, we constructed a risk assessment model to predict the recurrence of LUSC patients.

**Methods:**

RNA sequencing data (including miRNAs, lncRNAs, and mRNAs) and relevant clinical information were obtained from The Cancer Genome Atlas (TCGA) database. The differentially expressed lncRNAs, miRNAs, and mRNAs were identified using the “DESeq2” package of the R language. Univariate Cox proportional hazards regression analysis and Kaplan-Meier curve were used to identify recurrence-related genes. Stepwise multivariate Cox regression analysis was carried out to establish a risk model for predicting recurrence in the training cohort. Moreover, Kaplan-Meier curves and receiver operating characteristic (ROC) curves were adopted to examine the predictive performance of the signature in the training cohort, validation cohort, and entire cohort.

**Results:**

Based on the TCGA database, we analyzed the differentially expressed genes (DEGs) among 27 patients with recurrent stage I-IIIA LUSC and 134 patients with non-recurrent stage I-IIIA LUSC, and identified 431 lncRNAs, 36 miRNAs, and 746 mRNAs with different expression levels. Out of these DEGs, the optimal combination of DEGs was finally determined, and a nine-joint RNA molecular signature was constructed for clinical prediction of recurrence, including LINC02683, AC244517.5, LINC02418, LINC01322, AC011468.3, hsa-mir-6825, AC020637.1, AC027117.2, and SERPINB12. The ROC curve proved that the model had good predictive performance in predicting recurrence. The area under the curve (AUC) of the prognostic model for recurrence-free survival (RFS) was 0.989 at 3 years and 0.958 at 5 years (in the training set). The combined RNA signature also revealed good predictive performance in predicting the recurrence in the validation cohort and entire cohort.

**Conclusions:**

In the present study, we constructed a nine-joint RNA molecular signature for recurrence prediction of stage I-IIIA LUSC. Collectively, our findings provided new and valuable clinical evidence for predicting the recurrence and targeted treatment of stage I-IIIA LUSC.

## Introduction

Globally, lung cancer contributes greatly to the high incidence and mortality of cancer. Lung squamous cell carcinoma (LUSC) accounts for 20–30% of non-small cell lung cancer (NSCLC) cases, and it is the second most common pulmonary malignancy worldwide (Chen et al., 2017; Bray et al., 2018; Gao et al., 2019). When patients are diagnosed with stage I-IIIA LUSC, the standard treatment for patients is radical resection. Besides, the treatment of stage IIIA LUSC consists of either concurrent chemoradiation or surgery with perioperative chemotherapy with or without thoracic radiotherapy resection (Hirsch et al., 2017; Kamigaichi et al., 2019; Leroy et al., 2019). Patients with LUSC beyond stage IIIA are in the advanced stage of the disease, and usually no longer undergo surgical treatment. Moreover, radiotherapy and chemotherapy for these pateints are more conservative (Ettinger et al., 2017). Most recently, despite progress has been made in the diagnosis and treatment, the recurrence rate is still high (Kataoka et al., 2019). Recurrence is the most common cause of treatment failure in LUSC patients and the main obstacle to long-term survival (Dziedzic et al., 2016; Zhang et al., 2018). Moreover, 10–60% of patients with stage IA–IIIA LUSC develop relapse within 5 years after surgical resection (Chen et al., 2019; Kataoka et al., 2019). Therefore, evaluating the risk of postoperative recurrence for patients with stage I-IIIA is of great significance for guiding clinical treatment.

Recently, a large number of studies have used abnormal molecular expression to predict the prognosis of cancer patients, including long non-coding RNAs (lncRNAs), microRNAs (miRNAs), and messenger RNA (mRNAs). LncRNAs are a class of RNA transcripts with more than 200 nucleotides in length. Generally speaking, lncRNAs exhibit a wide range of regulatory activities without the coding capacity of proteins or peptides (Chi et al., 2019). MiRNAs are very short (20–24 nucleotides) non-coding RNAs first discovered in 1993 (Lee et al., 1993). They play a role in tumorigenesis by regulating cell apoptosis, metabolism, metastasis, cycle, and angiogenesis (Iqbal et al., 2019). Moreover, mRNAs are a class of essential biological macromolecules, which carry genetic information from the nucleus to the cytoplasm for the expressions of functional proteins. Abnormal expression of proteins can cause many diseases, such as cancer and genetic diseases (Li et al., 2019). Increasing evidence has demonstrated that these molecules can not only act as oncogenes or tumor suppressor genes but also participate in the occurrence and development of tumors through a complex mutual regulatory network (Mirgayazova et al., 2019). Besides, their expressions are tumor-tissue specific and have the potential as diagnostic or prognostic markers (Sim et al., 2018; Hou et al., 2020; Peng et al., 2020). Therefore, it is urgently needed to investigate the integration of multiple RNA expression profiles to identify molecular signatures.

In the present study, the data of lncRNAs, miRNAs, and mRNAs of patients with stage I-IIIA LUSC were simultaneously analyzed. We screened differentially expressed genes (DEGs) and established a prognostic model based on a nine-joint RNA molecular signature to predict the recurrence of stage I-IIIA. Clinically, our newly identified signature could predict the possibility of postoperative recurrence for patients with stage I-IIIA LUSC.

## Materials and Methods

### Data Retrieval

On November 15, 2020, the RNA sequencing data (including lncRNAs, miRNAs, and mRNAs) and clinical data of LUSC patients were obtained from The Cancer Genome Atlas (TCGA) database.^1^ We searched for the lncRNA and mRNA profiles of LUSC in TCGA datasets. The search formula was set to: click “cases,” Primary Site is bronchus and lung, Program Name is TCGA, Project ID is TCGA-LUSC, Workflow Type is HTseq-Counts, Data Category is transcriptome profiling, Data Type is Gene Expression Quantification, and Experimental Strategy is RNA-Seq. We obtained lncRNA and mRNA datasets of LUSC. We searched for the miRNA profiles of LUSC in TCGA datasets. The search formula was set to: click “cases,” Primary Site is bronchus and lung, Program Name is TCGA, Project ID is TCGA-LUSC, Data Category is transcriptome profiling, Data Type is miRNA Expression Quantification, and Experimental Strategy is miRNA-Seq. We obtained miRNA datasets of LUSC. Download path of clinicopathological information of LUSC was as follows; click “cases,” Primary Site is bronchus and lung, Program Name is TCGA, Project ID is TCGA-LUSC, and Data Category is clinical. We obtained the clinicopathological data of LUSC. We conducted a careful screening and analysis of the downloaded data. There were 504 patients in the TCGA-LUSC cohort, 170 of which had clear recurrence information. Finally, 27 patients with recurrent stage I-IIIA LUSC and 134 patients with non-recurrent stage I-IIIA LUSC were selected in this study. The inclusion criteria were set as follows: 1. patients with stage I-IIIA LUSC; 2. patients whose database contained three sequences of lncRNAs, mRNAs, and miRNAs; and 3. patients with complete clinical-pathological parameters. Normalized RNASeq data and clinical data were matched according to patient barcodes. The Chi-square test was used to analyze the correlation of clinical-pathological parameters between the data cohorts.

### Analysis of DEGs

By comparing the patients with recurrent stage I-IIIA LUSC to the patients with non-recurrent stage I-IIIA LUSC, the screening criteria of | log2 Fold Change | > 1, and *P* value <0.05 were used to identify significant lncRNAs, mRNAs, and miRNAs. All data in this study were obtained from the TCGA database, and ethical consent was not required.

### Identification of Recurrence-Associated DEGs

Using the “survival” and “survminer” packages of the R software, the recurrence-related DEGs were initially screened through univariate Cox regression analysis. The Kaplan-Meier survival curve was further drawn for the above-mentioned recurrence-related DEGs, and log-rank test *P* value <0.05 was adopted to clarify their relationship with the recurrence-free survival (RFS) of stage I-IIIA LUSC.

### Selection of the Optimal Combination of DEGs and Establishment of the Recurrence Risk Model

Univariate Cox regression analysis and log-rank test *P* value were combined to further screen out recurrence-related DEGs. Patients with stage I-IIIA LUSC were randomly divided into the training cohort and validation cohort through the random serial number generated by the computer. Subsequently, a stepwise multivariate Cox’s proportional hazards regression model was used to evaluate the relative contribution of DEGs in the training cohort to recurrence prediction and identify gene-based prognostic signature with independent prognostic value. Based on the expressions of genes included in the constructed signature and the corresponding coefficients, the risk score of each sample was calculated using the formula as follows:

Risk⁢score=expression⁢of⁢gene⁢ 1×β⁢1+expression⁢of⁢gene⁢ 2×β⁢2+⋯+expression⁢of⁢gene⁢n×β⁢n

Using the median risk score as the cutoff point, LUSC patients were separated into high- (> median risk score) and low-risk (< median risk score) subgroups.

### Evaluation of the Prognostic Model

Kaplan-Meier analysis was used to evaluate the RFS difference between low- and high-risk groups. A *P*-value <0.05 was considered statistically significant. The receiver operating characteristic (ROC) curve was performed to assess the effectiveness of the risk scoring model. The area under the curve (AUC) value was calculated based on the ROC curve. Additionally, the performance of this model was evaluated in the validation cohort as well as the entire cohort.

## Results

### Identification of the Recurrence-Associated DEGs From Stage I-IIIA LUSC

**FIGURE 1:**
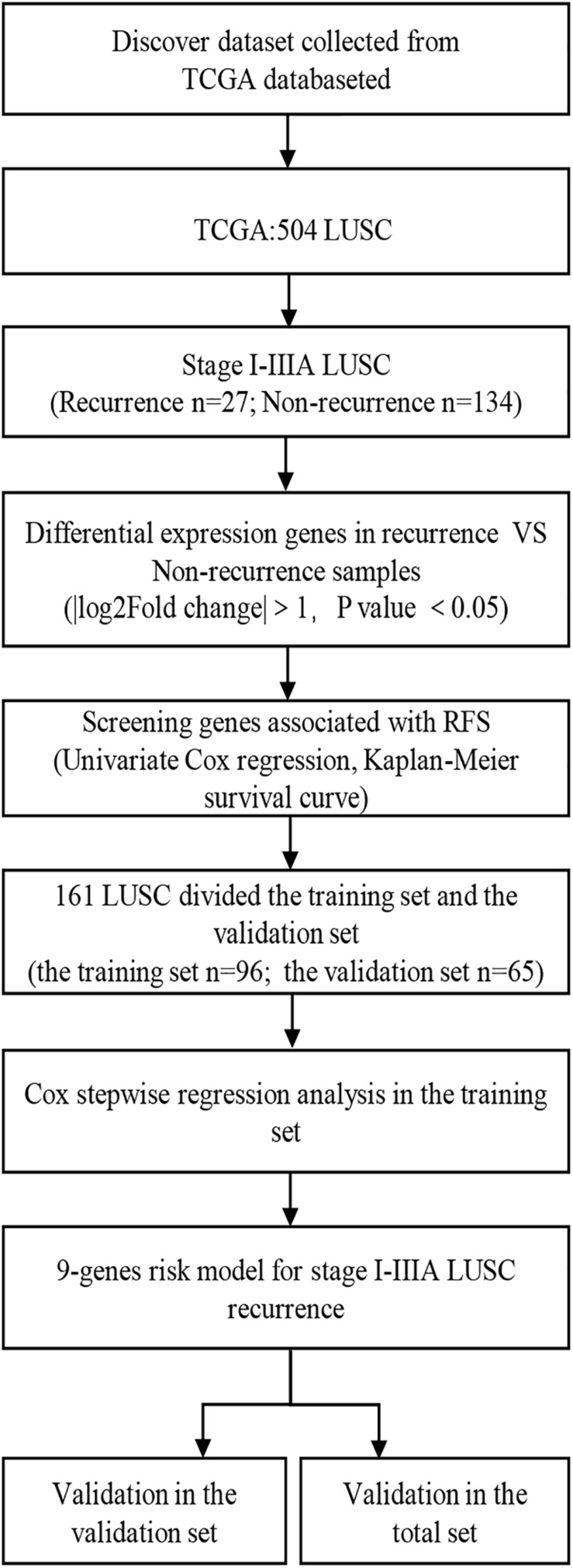
Overall design of the present study. LUSC, lung squamous cell carcinoma; RFS, Recurrence-free survival.

A total of 161 I-IIIA LUSC samples with RNA expression profiles (including lncRNAs, miRNAs, and mRNAs) and their clinical-pathological data were obtained from the TCGA database, including 27 recurrent samples and 134 non-recurrent samples. Figure 1 presents the flow chart of the study procedure. Through the “DESeq2” package, we identified 431 lncRNAs, 36 miRNAs, and 746 mRNAs with different expression levels. Supplementary Tables 1–3 show the identified DEGs.

Univariate Cox regression analysis was performed to identify the association of the DEGs with recurrence in LUSC patients. Using the cut-off threshold of Cox *P* < 0.05, a set of 46 lncRNAs, four miRNAs, and 48 mRNAs were selected (Supplementary Tables 4–6). Furthermore, the Kaplan-Meier survival curve was drawn for the above-mentioned recurrence-related DEGs to determine the relationship with the RFS of patients with stage I-IIIA LUSC, and 91 RNA molecules were identified as candidates, including 46 lncRNAs (up-regulated genes: *n* = 23; down-regulated genes: *n* = 23), four miRNAs (up-regulated genes: *n* = 3; down-regulated genes: *n* = 1), and 41 mRNAs (up-regulated genes: *n* = 11; down-regulated genes: *n* = 30) (Supplementary Table 7).

### Construction of Recurrence Risk Prediction Model

A total of 161 patients (recurrence = 27; non-recurrence = 134) of stage I-IIIA LUSC with and without recurrence were randomly assigned to the training cohort (*n* = 96; recurrence = 18; non-recurrence = 78) and validation cohort (*n* = 65; recurrence = 9; non-recurrence = 56). There was no statistical difference in age and gender between the two groups. Table 1 summarizes their clinical parameters.

**TABLE 1:** Clinical baseline characteristics of 161 patients with recurrence of stage I-IIIA LUSC.

Variables	Total	Recurrence	Recurrence-free	*t*/*x*^2^	*P*
**Gender**				0.8089	0.3684
Male	48 (29.8)	10 (37.0)	38 (28.4)		
Female	113 (71.2)	17 (63.0)	96 (71.6)		
**Age (y)**				0.2321	0.63
<=55	16 (9.9)	2 (7.4)	14 (10.4)		
>55	145 (90.1)	25 (92.6)	120 (89.6)		
**Pathologic stage**				10.8182	0.0045
Stage I	69 (42.9)	8 (29.6)	61 (45.5)		
Stage II	70 (43.4)	10 (37.0)	60 (44.8)		
Stage IIIA	22 (13.7)	9 (33.3)	13 (9.7)		
**M**				0.0308	0.1788
M0	111 (68.9)	19 (70.4)	92 (68.7)		
MX	50 (31.1)	8 (29.6)	42 (31.3)		
**N**				13.4839	0.0002
0	106 (65.8)	11 (40.7)	95 (70.9)		
1	43 (26.7)	11 (40.7)	32 (23.9)		
2	11 (6.8)	4 (14.8)	7 (5.2)		
X	1 (0.6)	1 (3.7)	0 (0)		
**T**				10.2132	0.0005
1	36 (22.4)	2 (7.4)	34 (25.4)		
2	89 (55.3)	15 (55.6)	74 (55.2)		
3	35 (21.7)	9 (33.3)	26 (19.4)		
4	1 (0.6)	1 (3.7)	0 (0)		

**FIGURE 2:**
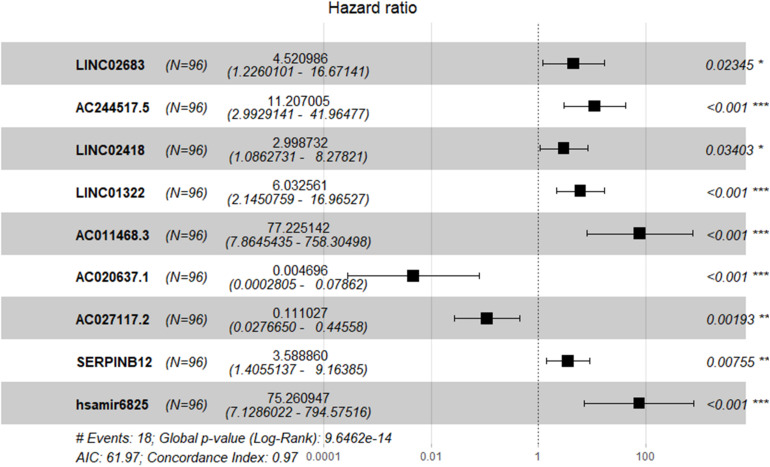
Expression profiles of nine genes for prediction of RFS in stage I-IIIA LUSC by multivariate Cox regression.

**FIGURE 3:**
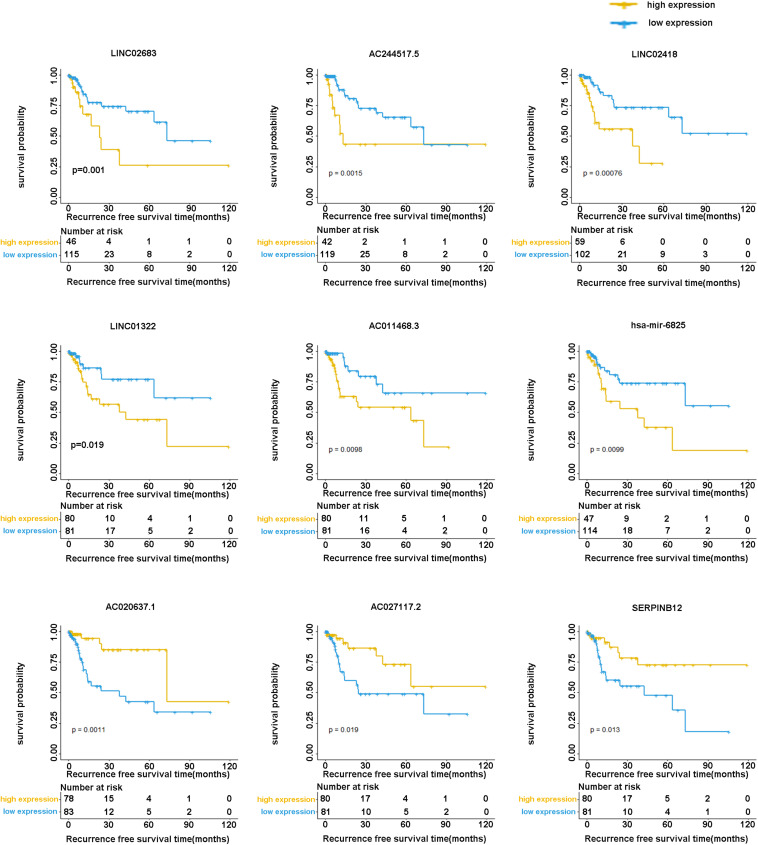
Kaplan-Meier analysis of RFS with nine genes (including LINC02683, AC244517.5, LINC02418, LINC01322, AC011468.3, hsa-mir-6825, AC020637.1, AC027117.2, and SERPINB12) in stage I-IIIA LUSC.

The optimal combination of nine recurrence-related DEGs was determined in the training cohort using the stepwise multivariate Cox regression analysis, including LINC02683, AC244517.5, LINC02418, LINC01322, AC011468.3, hsa-mir-6825, AC020637.1, AC027117.2, and SERPINB12. Table 2 lists the general information of these nine DEGs. Figure 2 shows that all nine DEGs were independent influencing factors for the recurrence of patients with I-IIIA LUSC. Kaplan-Meier survival analysis further revealed that the RFS of patients in the high expression group of LINC02683, AC244517.5, LINC02418, LINC01322, AC011468.3, and hsa-mir-6825 was lower compared with the low expression group. The RFS of patients in the low expression group of AC020637.1, AC027117.2, and SERPINB12 was significantly lower compared with the high expression group (Figure 3). These nine joint RNA molecules constituted a model, and the formula for prognostic risk score was as follows:

**TABLE 2:** Nine genes significantly related to the RFS of stage I-IIIA LUSC.

Gene name	coef	Type	Down/up -regulated	HR	95% CI	*p*-value
LINC02683	0.975	Risky	Up	2.650	1.218–5.764	0.011
AC244517.5	1.218	Risky	Up	3.381	1.526–7.492	0.001
LINC02418	1.302	Risky	Up	3.678	1.636–8.265	0.001
LINC01322	0.952	Risky	Up	2.592	1.132–5.938	0.019
AC011468.3	1.046	Risky	Up	2.847	1.242–6.53	0.010
AC020637.1	−1.481	Protective	Down	0.227	0.086–0.602	0.001
AC027117.2	−0.957	Protective	Down	0.384	0.168–0.88	0.019
SERPINB12	−1.015	Protective	Down	0.362	0.158–0.831	0.013
hsa-mir-6825	0.967	Risky	Up	2.631	1.226–5.647	0.01

Risk⁢score=(1.509×expression⁢of⁢LINC02683)+(2.417×expression⁢of⁢AC244517⁢.5)+(1.098×expression⁢of⁢LINC02418)+(1.797×expression⁢of⁢LINC01322)+(4.347×expression⁢of⁢AC011468⁢.3)+(-5.361×expression⁢of⁢AC020637⁢.1)+(-2.198×expression⁢of⁢AC027117⁢.2)+(1.278×expression⁢of⁢SERPINB12)+(4.321×expression⁢of⁢hsa-mir-6825).

Based on the results of the multivariate Cox stepwise regression model and median risk score point, the patients in the training cohort could be divided into “high risk” and “low risk,” respectively.

### Predictability Assessment of the Recurrence Risk Prediction Model in the Training Cohort

**FIGURE 4:**
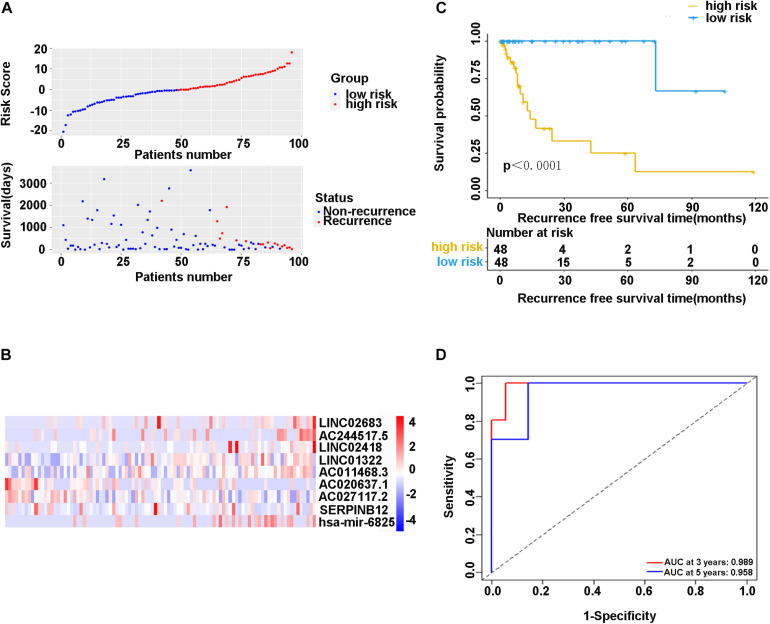
**(A)** Scatter diagram of the risk score and survival status of patients with stage I-IIIA LUSC in the training cohort. **(B)** The heatmap of nine-gene expression profiles for predicting recurrence risk model. **(C)** Kaplan-Meier plot showed significance between high-risk and low-risk patients in RFS by the prognostic model (*P* < 0.05). **(D)** The ROC curve analysis for the recurrence risk model. ROC, receiver operating characteristic; AUC, area under the ROC curve.

In the training cohort, the risk score and survival status of all patients are shown in Figure 4A. Patients in the high-risk group had higher risk scores and tended to have shorter RFS, and significantly more patients died in this group. The heatmap represented the expression profiles of the RFS model based on the risk scoring system (Figure 4B). The high-risk patients showed worse RFS compared with the low-risk group in the training cohort (χ^2^ = 8.842, *p* < 0.0001) (Figure 4C). There was only one patient with recurrence in the low-risk group (1.0%), while there were 17 patients with recurrence in the high-risk group (17.7%). Additionally, ROC curve analysis depicted the discriminative value of our established risk scoring system. We found that the AUC of the prognostic model for RFS was 0.989 at 3 years and 0.958 at 5 years (Figure 4D), indicating that the model had a good predictive performance. Taken together, these data demonstrated that the impressive RFS model based on the stepwise multivariate Cox regression analysis could be an effective method for predicting recurrence.

### Validation of the Nine-Gene Signature for Recurrence Risk Prediction Model

**FIGURE 5:**
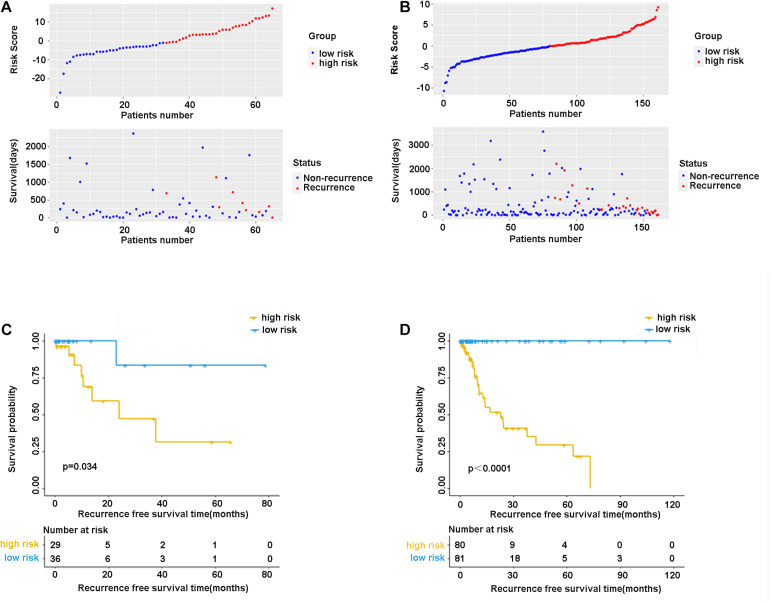
The distribution of risk score and survival status of the nine-gene signature in the validation cohort **(A)** and entire cohort. **(B)** Kaplan-Meier curves of overall survival in the validation cohort **(C)** and entire cohort **(D)**.

**FIGURE 6:**
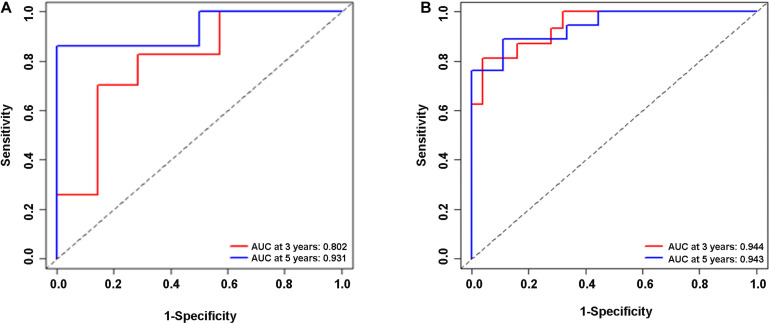
ROC analysis of the nine-gene risk model in the validation cohort **(A)** and the entire sample cohort **(B)**.

In the validation cohort (*n* = 65) and the entire sample cohort (*n* = 161), the RFS of patients in the high-risk group was generally lower compared with the low-risk group, and more patients in the high-risk group were likely to die (Figures 5A,B). Figure 5C shows that in the validation cohort, the median RFS in the high-risk group was significantly shorter compared with the lower-risk group (χ^2^ = 5.575, *P* = 0.034). In the entire sample concentration, the RFS of the high-risk group was lower compared with the low-risk group (χ^2^ = 8.642, *P* < 0.0001) (Figure 5D). The time-dependent ROC curve suggested that the model showed an equal predictive performance in the validation cohort (3-year AUC = 0.802, 5-year AUC = 0.931) and the entire cohort (3-year AUC = 0.944, 5-year AUC = 0.943) (Figures 6A,B). The above-mentioned results suggested that the nine-gene recurrence model could effectively distinguish the possibility of postoperative recurrence between high- and low-risk groups of patients with stage I-IIIA LUSC.

## Discussion

In the past 30 years, surgery alone has been the standard treatment for patients with stage I-IIIA NSCLC. Even with complete resection, the 5-year survival rates remain disappointing. Meanwhile, little progress has been made in reducing the recurrence and subsequent mortality of lung cancer, especially LUSC (Li et al., 2018; Nasim et al., 2019). According to previous reports, LUSC has a higher local recurrence rate compared with lung adenocarcinoma (Woody et al., 2017; Mcaleese et al., 2019). The recurrence risk of LUSC patients of stage IA, IB, IIA, IIB, and IIIA is 33.9, 37.8, 61.2, 57.9, and 62.8%, respectively (Consonni et al., 2015). Although IB patients have been considered for adjuvant therapy, the benefits for stage I patients are still controversial (Tsuboi et al., 2007). Meanwhile, patients with stage II-IIIA may also have a good prognosis since they neither need adjuvant therapy nor benefit from adjuvant therapy (Subramanian and Simon, 2010; Wu et al., 2017). Effective stratification and prediction of patient prognosis will help guide the treatment strategies. Recent studies have proved that molecular approaches are effective in predicting the prognosis of cancer patients, which can help optimize individualized treatment, such as breast cancer, and liver cancer (Wan et al., 2010; Gu et al., 2019). In the present study, patients with stage I-IIIA LUSC in the TCGA database were included as the research object. After a series of analyses, the nine-gene signature could effectively predict the recurrence risk of stage I-IIIA LUSC, which was beneficial to the clinical judgment of adjuvant treatment for stage I LUSC patients after surgery, avoiding excessive radiotherapy and chemotherapy for stage II and III patients with lower risk.

The combined analysis of a panel of multiple factors, rather than a single biomarker, will have more power to provide useful information for clinical practice. For example, by analyzing differential mRNAs, Sun et al. have found that S100A16, IGKV4, S100P, ANGPTL4, SEMA4B, and LGR4 are high-risk immune genes in NSCLC that affect the prognosis of the disease (Sun et al., 2020). Sim et al. have found that three miRNAs (let-7g-5p, miR-143-3p, and miR-374a-5p) are associated with postoperative recurrence of lung adenocarcinoma in TCGA datasets (Sim et al., 2018). We believed that an ideal prognostic classifier for LUSC recurrence risk should be robust and potentially feasible in clinical samples. Combined analysis of a variety of molecules to find the optimal combination of DEGs would overcome barriers to sample collection and storage. In our present study, lncRNAs, miRNAs, and mRNAs were included in the gene screening, and finally, a nine-gene signature related to tumor recurrence in LUSC patients was determined. Time-independent ROC analysis showed that our nine-gene signature was effective for prognostic evaluation, suggesting that the model could be used in the training cohort (3-year AUC = 0.989, 5-year AUC = 0.958), the validation cohort (3-year AUC = 0.802, 5-year AUC = 0.931), and the entire data cohort (3-year AUC = 0.944, 5-year AUC = 0.943). The Kaplan-Meier survival charts of the three groups also showed that the RFS of the high-risk group was significantly lower compared with the low-risk group.

Among the identified genes in the present study, we found that LINC02683, AC244517.5, LINC02418, LINC01322, AC011468.3, and hsa-mir-6825 were risk factors. In contrast, AC020637.1, AC027117.2, and SERPINB12 were protective factors. Multivariate analysis showed that these high-level risk factors and low-level protective factors were independent prognostic factors. These results were consistent with previous research showing that LINC02418 functions as an oncogene through regulating the miR-4677-3p/SEC61G axis to accelerate the progression of NSCLC (Han, 2019). There is also a literature reporting that high expression of LINC02418 in lung adenocarcinoma promotes the growth of cancer cells (Wang et al., 2020). Considering the protective factors, we noted that SERPINB12 is abnormally expressed in the lung in previous reports, while there is no further explanation for its role (Niehaus et al., 2015). SERPINB12, like some other intracellular serine protease inhibitors, may play an important role in the barrier function by providing epithelial cell protection (Askew et al., 2001). Another article has reported that SERPINB12 maybe related to the abnormal growth and dysfunction of ovarian cancer. It is a potential biomarker and can be used for the early detection of ovarian cancer in women (Jo et al., 2014). Other LINC02683, AC244517.5, LINC01322, AC011468.3, AC020637.1, AC027117.2, and hsa-mir-6825 were reported for the first time in LUSC in the present study.

However, this study also had certain limitations. First, the sample size of the TCGA database was limited, and the effectiveness of the nine-gene model established in this study still needs to be further confirmed by large-sample clinical studies. Second, the TCGA database is a human genome project jointly launched by the National Cancer Institute and the National Human Genome Institute. We need to further expand the subjects of different races and perform multi-center research.

Collectively, our study successfully created a nine-gene signature that could efficiently screen outpatients with a high risk of recurrence after radical surgery of stage I-IIIA LUSC. This signature was a powerful complement to the prognostic factors of LUSC. However, a series of clinical samples are still needed to further validate its prognostic value in LUSC patients.

## Data Availability Statement

The datasets presented in this study can be found in online repositories. The names of the repository/repositories and accession number(s) can be found in the article/Supplementary Material.

## Author Contributions

LS and JL designed the study, performed the data analysis, and wrote the manuscript. XL, XY, and SZ acquired the data and carried out the bioinformatic analysis. XW, NW, and KX collected important background information and carried out a literature search. XJ and YZ drafted the manuscript. All authors read and approved the final manuscript.

## Conflict of Interest

The authors declare that the research was conducted in the absence of any commercial or financial relationships that could be construed as a potential conflict of interest.

## Footnotes

1 https://www.cancer.gov/about-nci/organization/ccg/research/structural-genomics/tcga
