# Supplementary material for: A Combined RNA Signature Predicts Recurrence Risk of Stage I-IIIA Lung Squamous Cell Carcinoma
Source: Front Genet. 2021 Jun 14;12:676464. doi: 10.3389/fgene.2021.676464 (PMC8236863; doi:10.3389/fgene.2021.676464)
Supplement: Supplementary file 1 [file Table_1.DOCX]

**Supplementary Table 1**: Differentially expressed lncRNAs

| ID | baseMean | log2FoldChange | pvalue | padj |
| --- | --- | --- | --- | --- |
| FAM41C | 6.016991508 | -1.78588831 | 0.006958107 | 0.232960671 |
| AL590822.1 | 2.26614473 | -1.01141841 | 0.021175969 | 0.355128982 |
| AL034417.2 | 9.430212919 | 1.009023538 | 0.002048328 | 0.126430311 |
| LINC01714 | 1.419386941 | 1.315958454 | 0.026754033 | 0.391499136 |
| AL357552.2 | 0.517346799 | -1.146654913 | 0.04230615 | NA |
| Z98257.1 | 25.4674758 | 2.188450885 | 7.95E-06 | 0.00486436 |
| LINC01778 | 1.903588821 | 1.030036626 | 0.03265941 | 0.413643904 |
| AL139147.1 | 4.951166181 | 1.011265097 | 0.010969897 | 0.285017546 |
| LINC01788 | 0.546261774 | 1.831167019 | 0.005031539 | NA |
| AL031429.2 | 0.626417602 | -1.370838518 | 0.005864796 | NA |
| AC091614.1 | 0.953711608 | 2.17985636 | 4.96E-07 | 0.000472164 |
| AC114485.1 | 1.974667768 | -2.590655002 | 0.031042427 | 0.408074603 |
| LINC01160 | 43.59875111 | -1.030819279 | 0.006430359 | 0.225849346 |
| AL589765.7 | 1.066477123 | -1.757670347 | 0.022743057 | 0.37043645 |
| AL161636.1 | 5.201930608 | -1.26950688 | 0.019850598 | 0.352986457 |
| AL365181.4 | 1.735017874 | -1.351974485 | 0.034705108 | 0.421792382 |
| LINC00970 | 7.661781847 | -1.350176256 | 0.019225934 | 0.347648696 |
| AL133553.1 | 1.416851833 | -1.11213794 | 0.018959438 | 0.347648696 |
| AL023754.1 | 25.99601679 | -1.088816939 | 0.018522632 | 0.342888731 |
| LINC01740 | 1.701432436 | -1.072255042 | 0.039945331 | 0.430167611 |
| LINC02474 | 1.360408514 | -1.285986791 | 0.046767711 | 0.454604257 |
| LINC02813 | 0.716776555 | -1.440138081 | 0.017568131 | NA |
| ITPKB-IT1 | 1.831580139 | -1.012541824 | 0.010528038 | 0.281108448 |
| AL160408.5 | 6.090990132 | -1.306234026 | 0.00408307 | 0.182053649 |
| AL160408.1 | 20.00294037 | -1.006773658 | 0.008321009 | 0.253845906 |
| AL160408.4 | 9.769105317 | -1.039776387 | 0.041173461 | 0.436681449 |
| KIF26B-AS1 | 3.037524162 | -1.074258227 | 0.020387552 | 0.355128982 |
| SNTG2-AS1 | 25.53721371 | 1.118081767 | 0.039127454 | 0.430167611 |
| LINC01250 | 4.029069072 | 1.537853977 | 0.000432598 | 0.056984035 |
| LINC00487 | 5.520543188 | 1.044315755 | 0.004674642 | 0.193138263 |
| MIR3681HG | 8.770808195 | -1.089081973 | 0.012183669 | 0.294093143 |
| AC064875.1 | 4.871592948 | -1.174835578 | 0.00645585 | 0.225849346 |
| AC008278.2 | 0.966487068 | -1.636893125 | 0.014961106 | 0.315199427 |
| MYCNOS | 8.895630866 | -1.285379861 | 0.027913996 | 0.394235341 |
| AC012506.3 | 1.289833769 | 2.618347304 | 9.45E-07 | 0.000810251 |
| AC012506.2 | 0.90485987 | 1.377804675 | 0.048584252 | 0.456549622 |
| LINC00486 | 2.623121955 | -1.177421019 | 0.022188385 | 0.362932528 |
| LINC01805 | 4.327253934 | -1.852949743 | 0.00087279 | 0.08698465 |
| AC105053.1 | 1.114893289 | -1.190169207 | 0.025425039 | 0.383658469 |
| AC103563.7 | 3.104573689 | -1.419885306 | 0.008322331 | 0.253845906 |
| AC092667.1 | 3.231934398 | -1.962635943 | 0.005832609 | 0.21497409 |
| AC108868.1 | 4.091646586 | -3.080926167 | 0.002194203 | 0.132440237 |
| AC007556.1 | 1.378137916 | -1.177964039 | 0.024610401 | 0.379380833 |
| AC007277.1 | 2.990632806 | -1.29171612 | 0.0039687 | 0.182053649 |
| AC074286.1 | 1.503327132 | -1.400575671 | 0.009897197 | 0.274077549 |
| LINC01792 | 2.213956104 | 1.652852794 | 0.000949867 | 0.09045903 |
| MYOSLID | 115.7884493 | 1.072831718 | 0.000847969 | 0.086523096 |
| AC093843.1 | 1.561767794 | -1.187103086 | 0.014378762 | 0.310901453 |
| AC062015.1 | 4.263864215 | -2.036895092 | 0.009307293 | 0.264148364 |
| AC093802.2 | 1.921064561 | -2.482420531 | 0.034994392 | 0.422446389 |
| AC104809.2 | 9.236676033 | -1.174942021 | 0.006003181 | 0.21497409 |
| AC104809.1 | 5.072429998 | -1.706705669 | 0.005356501 | 0.206316751 |
| BOK-AS1 | 3.020685864 | 1.427786162 | 0.002939633 | 0.152700548 |
| AC034195.1 | 0.872766967 | -1.545457831 | 0.00420565 | 0.182053649 |
| AC087857.1 | 0.767261642 | 1.736605993 | 0.011407164 | 0.286290596 |
| AC077690.1 | 1.608958326 | -1.313085562 | 0.039094054 | 0.430167611 |
| SRGAP3-AS2 | 21.52148513 | -1.150923821 | 0.048511232 | 0.456549622 |
| HHATL-AS1 | 2.101473081 | -1.43066692 | 0.020776715 | 0.355128982 |
| AC121764.1 | 10.83098335 | -2.389854427 | 0.001474206 | 0.109873211 |
| LINC02005 | 7.625542387 | -1.608300124 | 0.008557385 | 0.257352109 |
| LINC00973 | 4.797113554 | 3.479125506 | 1.76E-12 | 1.50E-08 |
| LINC00488 | 2.220646701 | -2.658449413 | 0.014377268 | 0.310901453 |
| AC092894.1 | 1.822833838 | -2.207278362 | 0.014050347 | 0.310901453 |
| SIDT1-AS1 | 1.103027638 | -1.210467828 | 0.024175949 | 0.376354148 |
| ZBTB20-AS4 | 1.23501831 | -1.287880466 | 0.004714856 | 0.193138263 |
| AC016924.1 | 6.892036631 | 1.050718179 | 0.007550781 | 0.243299781 |
| LINC02046 | 2.289015201 | -2.028426507 | 0.034891004 | 0.421792382 |
| LINC02066 | 0.819125967 | -1.427750403 | 0.035255585 | 0.423808722 |
| LINC01324 | 1.731580948 | -2.058854493 | 0.039900613 | 0.430167611 |
| LINC01322 | 13.29140339 | 1.31940725 | 0.000132163 | 0.032364745 |
| NAALADL2-AS2 | 36.1962293 | -1.133120275 | 0.021403818 | 0.355788642 |
| AC125618.1 | 3.09831553 | -1.687940107 | 0.00022061 | 0.040782046 |
| LINC01206 | 1447.230785 | -2.632552078 | 2.67E-05 | 0.011435816 |
| AC084211.1 | 1.429007852 | -1.414684642 | 0.015772488 | 0.324801504 |
| P3H2-AS1 | 17.81428369 | -1.096575802 | 0.014421713 | 0.310901453 |
| ATP13A4-AS1 | 12.61038271 | -1.250176655 | 0.045151089 | 0.448424087 |
| AL645924.1 | 3.071714751 | -1.225118892 | 0.026146909 | 0.388397154 |
| LINC01587 | 7.602960414 | 1.126273615 | 0.001909763 | 0.124950982 |
| AC025539.1 | 12.76090856 | -1.168479858 | 0.001863029 | 0.122830908 |
| AC007370.2 | 0.702423305 | -1.187050074 | 0.014226066 | NA |
| AC097512.1 | 5.26017201 | -1.625459809 | 0.043603937 | 0.442924744 |
| AC104071.1 | 6.540771731 | -2.637498574 | 0.006636154 | 0.226392221 |
| LINC02506 | 5.005747426 | 2.405823015 | 0.00139823 | 0.106055164 |
| LINC01258 | 3.088598186 | -1.62703823 | 0.003898148 | 0.182053649 |
| AC239584.1 | 0.694617102 | 1.580643533 | 0.01742042 | NA |
| LINC01088 | 45.32243073 | -2.257912641 | 1.63E-08 | 4.58E-05 |
| PRKG2-AS1 | 0.315584739 | 2.297854259 | 0.047016105 | NA |
| AC139718.1 | 1.624646856 | -1.71476955 | 0.041366262 | 0.437103821 |
| AC004704.1 | 4.714684197 | -1.275342083 | 0.048108812 | 0.455733751 |
| LINC02516 | 5.116598217 | -1.202242814 | 0.000435737 | 0.056984035 |
| AC078850.2 | 0.936900975 | 1.517062499 | 0.000735561 | 0.080826792 |
| AC079380.1 | 1.680511729 | 1.233089482 | 0.020792677 | 0.355128982 |
| LINC02432 | 25.13068619 | -1.375022965 | 0.005146635 | 0.201423781 |
| AC109811.1 | 1.403169745 | 1.670588548 | 0.016844193 | 0.330272457 |
| LINC02266 | 1.594759184 | -1.374679186 | 0.045301128 | 0.448873956 |
| LINC02507 | 1.132654316 | -1.281881087 | 0.021255532 | 0.355128982 |
| AC110813.1 | 0.495537197 | 1.575430649 | 0.00177283 | NA |
| AC097375.1 | 0.783165503 | -1.120472743 | 0.042830798 | 0.44017119 |
| AC093599.1 | 7.605674002 | -1.148171174 | 0.01824928 | 0.340638784 |
| LINC02437 | 3.974045766 | -1.773137696 | 0.010114224 | 0.275203224 |
| LINC02515 | 0.579000733 | 2.439700539 | 0.008630898 | NA |
| DBET | 1.553122508 | 1.056796462 | 0.020786728 | 0.355128982 |
| LINC01511 | 3.93689595 | -1.293147483 | 0.01537293 | 0.321369236 |
| LSINCT5 | 1.475156653 | -1.187805169 | 0.034378947 | 0.421400842 |
| LINC01019 | 1.030645596 | 1.964048332 | 0.030438527 | 0.403852348 |
| CTD-2297D10.2 | 2.975907389 | -1.058122701 | 0.046403029 | 0.454043826 |
| AC092335.1 | 2.433900643 | -2.779243844 | 0.021373735 | 0.355788642 |
| LINC02100 | 11.22784611 | 1.156054626 | 0.000214229 | 0.040782046 |
| AC093274.1 | 1.608916481 | -1.23011793 | 0.02369579 | 0.375409636 |
| LINC02211 | 5.291452366 | -1.14024743 | 0.037843609 | 0.429888826 |
| AC010457.1 | 4.261817445 | 1.708662651 | 0.000774997 | 0.082006143 |
| LINC00603 | 3.497434569 | -2.661615078 | 0.034751506 | 0.421792382 |
| LINC01033 | 6.556087602 | -1.927038105 | 0.000786978 | 0.082258425 |
| LINC02122 | 3.016762744 | -1.55375893 | 0.03378391 | 0.418442047 |
| AC093281.2 | 1.325672471 | 1.462267758 | 0.004972502 | 0.197966078 |
| AC114316.1 | 10.28627641 | 1.134251967 | 0.007727922 | 0.245663243 |
| LINC02234 | 2.42908213 | -1.116910844 | 0.012832225 | 0.298872273 |
| AC010255.1 | 2.68656931 | -1.102653621 | 0.021183134 | 0.355128982 |
| AC244517.5 | 0.692047277 | 1.692086356 | 0.018085083 | NA |
| AC244517.12 | 1.003191573 | 1.113050775 | 0.033587646 | 0.417710224 |
| AC008700.1 | 0.572407472 | 1.159984741 | 0.017186506 | NA |
| STK32A-AS1 | 4.335384231 | 1.638960427 | 0.005803092 | 0.21497409 |
| AC025434.1 | 1.591616311 | -1.601367716 | 0.011649993 | 0.288589853 |
| AL033381.2 | 3.214224823 | -1.429994931 | 0.008888526 | 0.262759044 |
| AL033381.1 | 4.75467114 | -1.806244615 | 0.000438799 | 0.056984035 |
| LINC02521 | 1.356234109 | -1.032755878 | 0.045146787 | 0.448424087 |
| SERPINB9P1 | 80.00030425 | 1.105886358 | 0.000110967 | 0.028821201 |
| LINC02525 | 2.346046767 | -1.932860781 | 0.043278223 | 0.441851033 |
| LINC01108 | 10.24846536 | -1.20056393 | 0.011559038 | 0.287166706 |
| AL513188.1 | 0.670828041 | 1.08379004 | 0.013903439 | NA |
| TDRG1 | 2.75309775 | -1.539587235 | 0.032768363 | 0.4142443 |
| AL391807.1 | 8.764099727 | 1.132455444 | 0.005012092 | 0.197966078 |
| AL445465.1 | 1.00632178 | 1.924619679 | 0.000259284 | 0.042736956 |
| LINC02535 | 7.824697769 | 1.285911013 | 0.001343991 | 0.10568211 |
| AL132996.1 | 0.56056081 | -1.043806228 | 0.041334138 | NA |
| LINC02518 | 8.358582731 | -1.101660736 | 0.01308905 | 0.303206082 |
| AL445224.1 | 1.35644734 | -1.473927108 | 0.026901906 | 0.391939024 |
| TRDN-AS1 | 0.393038776 | 1.146447201 | 0.049107783 | NA |
| CT69 | 129.5105311 | -1.026457755 | 0.023377618 | 0.375152352 |
| MYB-AS1 | 1.029174076 | 1.696454575 | 0.004026873 | 0.182053649 |
| AL355312.3 | 31.83830975 | 1.853703139 | 7.09E-06 | 0.004675589 |
| AL049820.1 | 0.461494615 | 1.304095527 | 0.016178667 | NA |
| AL139393.2 | 3.331434344 | 1.243888949 | 0.04251198 | 0.437944929 |
| HGC6.3 | 3.845383558 | -1.450092089 | 0.008994523 | 0.262759044 |
| AL606970.4 | 6.376587163 | -1.409925054 | 0.04211373 | 0.437186986 |
| AC093627.1 | 2.876463366 | -1.414952615 | 0.040798721 | 0.435474268 |
| AC093627.7 | 3.834910908 | -1.279248374 | 0.002292219 | 0.135769054 |
| AC147651.1 | 4.911806283 | -1.932760268 | 0.001949094 | 0.12560667 |
| HRAT92 | 64.51580593 | -1.26882951 | 3.38E-05 | 0.012578745 |
| ELFN1-AS1 | 36.49486594 | -1.165170935 | 0.014774857 | 0.315199427 |
| AC019117.2 | 15.99677238 | -1.47263134 | 0.001980895 | 0.125764843 |
| AC005100.1 | 2.761508165 | -1.841657478 | 0.044339086 | 0.44317683 |
| AC004947.1 | 4.693911322 | -1.635265411 | 0.000676155 | 0.079528685 |
| AC008080.4 | 2.355904188 | -1.36960627 | 0.001167361 | 0.098392955 |
| AOAH-IT1 | 2.149176798 | -1.101930896 | 0.037040528 | 0.429888826 |
| AC023669.1 | 3.016133379 | -1.352673728 | 0.031705222 | 0.410112666 |
| AC004870.2 | 8.659009753 | -1.931559439 | 0.005911461 | 0.21497409 |
| CACNA2D1-AS1 | 0.84938641 | 1.422298813 | 0.024194509 | 0.376354148 |
| AC004522.3 | 1.058256452 | 1.152884359 | 0.043699283 | 0.442924744 |
| AC005050.1 | 2.939753261 | -1.270086968 | 0.006005848 | 0.21497409 |
| AC002066.1 | 6.624420105 | 1.072969779 | 0.00099779 | 0.091892904 |
| AC018635.2 | 1.175678908 | -1.117207749 | 0.0161834 | 0.327714191 |
| AC009275.1 | 26.77072597 | 1.089662012 | 0.000210274 | 0.040782046 |
| WDR86-AS1 | 75.21357677 | -1.317006282 | 0.000328867 | 0.050228683 |
| AC006372.3 | 2.264874088 | 1.611488166 | 0.000310126 | 0.048328924 |
| AC006372.1 | 1.997167796 | 1.424365155 | 0.039734522 | 0.430167611 |
| AC011899.1 | 1.574175822 | -1.979781802 | 0.014857837 | 0.315199427 |
| AC110995.1 | 8.507988438 | 1.459463765 | 9.90E-05 | 0.026524583 |
| LINC01203 | 0.974296613 | 1.419072313 | 0.013780484 | 0.310850603 |
| AC003035.1 | 1.897799644 | -1.014104514 | 0.02674988 | 0.391499136 |
| AL035425.3 | 170.3972989 | -1.383907262 | 0.031850516 | 0.411130685 |
| RHOXF1-AS1 | 285.4353043 | -1.252578281 | 0.010332347 | 0.277709193 |
| SPANXA2-OT1 | 1.390587806 | 1.724058936 | 0.001522371 | 0.112484848 |
| AF131215.7 | 15.44643925 | 1.090597541 | 0.003212839 | 0.161632946 |
| FAM167A-AS1 | 7.026834625 | -1.558987443 | 0.00709161 | 0.236506583 |
| AC027117.2 | 4.170767715 | -1.031363323 | 0.000753317 | 0.081137789 |
| AC055854.1 | 7.720307272 | -1.031689852 | 0.028911727 | 0.399334167 |
| AC037441.1 | 5.273561448 | -1.377543178 | 0.019205166 | 0.347648696 |
| AC120193.1 | 6.734658063 | -1.442392577 | 0.00251999 | 0.142097573 |
| AC025871.1 | 0.637016828 | 1.058700595 | 0.031423259 | NA |
| AC087623.1 | 65.62507827 | -1.119377821 | 0.00253911 | 0.142239924 |
| AC069120.1 | 9.099851006 | -1.317090984 | 0.026008832 | 0.387117676 |
| AC044893.1 | 6.159430338 | -4.077644569 | 1.13E-06 | 0.000883111 |
| AC009646.2 | 1.881610669 | -2.086374421 | 0.008661213 | 0.258659421 |
| AC131902.1 | 2.187526045 | -2.014069969 | 0.001026416 | 0.092604317 |
| AC022034.3 | 4.480918366 | -2.257201968 | 2.58E-05 | 0.011435816 |
| AC012349.1 | 3.20298441 | -1.202823149 | 0.036097896 | 0.427440343 |
| LINC00968 | 16.82687776 | -1.498553453 | 2.42E-05 | 0.011435816 |
| AC090136.3 | 6.185906972 | -1.076213105 | 0.000724258 | 0.080618364 |
| AC083967.1 | 2.252667053 | 1.910029201 | 0.000165613 | 0.036396673 |
| AC022893.2 | 18.94219751 | -1.884495131 | 0.006720374 | 0.227044888 |
| LINC01419 | 58.87767459 | 3.448136166 | 3.22E-05 | 0.012546815 |
| AC100801.1 | 6.727378281 | -2.13460265 | 0.003660639 | 0.174307449 |
| AC107909.1 | 0.629616464 | 1.22004919 | 0.007908947 | NA |
| AP001574.1 | 13.9286811 | -1.431613185 | 4.89E-05 | 0.014808301 |
| AC079061.1 | 2.571668109 | -1.224935788 | 0.02117464 | 0.355128982 |
| LINC00536 | 4.825704715 | -1.144188972 | 0.039931047 | 0.430167611 |
| AC100872.2 | 2.80992743 | -1.948697864 | 0.00081542 | 0.084204443 |
| FAM83A-AS1 | 174.230769 | 1.478410658 | 0.001935585 | 0.12560667 |
| AC090192.2 | 3.069980529 | 1.603777485 | 0.001170935 | 0.098392955 |
| AC110741.1 | 2.595810779 | 1.082585658 | 0.006656264 | 0.226392221 |
| NCRNA00250 | 1.007066209 | -1.123608092 | 0.013818 | 0.310850603 |
| AC046195.1 | 17.8642748 | -1.730676741 | 0.001647081 | 0.114807236 |
| AC011676.1 | 7.489496728 | 1.016450785 | 5.84E-05 | 0.016692974 |
| LINC01230 | 1.514772047 | 1.613953838 | 0.000717858 | 0.080618364 |
| LINC02843 | 3.706366567 | -3.507092713 | 0.006019575 | 0.21497409 |
| AL354754.1 | 16.52192295 | -2.128371411 | 0.000279598 | 0.045215706 |
| TTLL11-IT1 | 4.786509129 | 1.739720658 | 1.30E-05 | 0.007436948 |
| AL161785.1 | 34.56962241 | 1.21582621 | 0.000362253 | 0.052624941 |
| PRRX2-AS1 | 0.931798094 | -1.062194226 | 0.03548493 | 0.424778401 |
| GPRACR | 4.169824045 | 1.639587726 | 0.000144695 | 0.033956824 |
| AL669970.3 | 0.973734126 | -1.372128956 | 0.040128726 | 0.431006651 |
| AL390778.2 | 25.09889904 | -1.390781866 | 0.028642511 | 0.39788487 |
| LINC02688 | 19.80535913 | 1.218688696 | 0.001275925 | 0.104151966 |
| H19 | 3490.283936 | 1.086526162 | 0.028159847 | 0.394235341 |
| LINC02683 | 0.581090719 | 2.001314734 | 0.001210651 | NA |
| AC009549.1 | 19.74717369 | 2.078018542 | 1.51E-07 | 0.000215623 |
| AC110058.1 | 3.171825469 | -2.813323056 | 0.000212632 | 0.040782046 |
| MPPED2-AS1 | 5.617510689 | -1.663090153 | 0.007652151 | 0.244726065 |
| AC044839.1 | 4.221206753 | -1.468995012 | 0.037002118 | 0.429888826 |
| AC044839.2 | 3.453510551 | -2.56032908 | 0.003202522 | 0.161632946 |
| AP001636.3 | 16.86525253 | 1.666693059 | 0.000128426 | 0.032364745 |
| AP003108.1 | 0.930716513 | 2.357249841 | 4.73E-06 | 0.003381531 |
| OR7E11P | 1.16796084 | 1.28680947 | 0.00731775 | 0.238343815 |
| AP000439.2 | 4.382032524 | -1.32048913 | 0.043303566 | 0.441851033 |
| AP003555.2 | 3.613770918 | -1.094605831 | 0.032978107 | 0.416281815 |
| AP000851.2 | 9.80270248 | -1.857970608 | 0.00100771 | 0.091892904 |
| AP002008.3 | 2.068976395 | 1.023015631 | 0.025271875 | 0.382019824 |
| AP000842.2 | 0.956395367 | -1.720509272 | 0.033625144 | 0.417710224 |
| KIRREL3-AS1 | 2.676383718 | -1.461913718 | 0.02940986 | 0.399553766 |
| AP001783.1 | 6.347564944 | -1.931275143 | 0.000757324 | 0.081137789 |
| LINC02873 | 3.394443432 | 1.400268971 | 0.035095443 | 0.423070388 |
| LINC02551 | 16.32257463 | 1.030393734 | 0.018852874 | 0.346755328 |
| AP000844.2 | 1.475964004 | 2.273055345 | 0.00288435 | 0.151467847 |
| LINC00200 | 1.699785196 | 4.063640362 | 2.87E-05 | 0.011696841 |
| PROSER2-AS1 | 18.6074499 | 1.162302872 | 3.47E-07 | 0.000371935 |
| ITGB1-DT | 6.765963871 | 1.438784785 | 2.51E-07 | 0.000307297 |
| LINC01518 | 34.64235758 | -1.713294414 | 0.01378517 | 0.310850603 |
| AL358394.2 | 1.915369892 | -1.492287795 | 0.013201783 | 0.303643316 |
| AL358394.1 | 11.72786195 | -1.142151078 | 0.045742265 | 0.451734464 |
| AC245041.1 | 189.1341855 | 1.016473263 | 0.006120543 | 0.217672913 |
| LINC02672 | 1.363083521 | -2.293146011 | 0.021182398 | 0.355128982 |
| PLCE1-AS1 | 2.192796056 | -1.229648325 | 0.005093699 | 0.20026649 |
| LINC01475 | 6.40056547 | -1.645882967 | 0.020201589 | 0.355128982 |
| TLX1NB | 3.566753999 | -1.783402679 | 0.019795566 | 0.352842816 |
| LINC00601 | 4.449260328 | 1.737029549 | 0.001310596 | 0.104983505 |
| LINC01163 | 5.904928093 | -1.585790366 | 0.003330296 | 0.164045804 |
| AL161645.1 | 5.512827213 | -1.274514033 | 0.004161124 | 0.182053649 |
| AC026369.3 | 11.87373572 | -1.260175812 | 0.004461462 | 0.187447002 |
| AC004672.1 | 1.221853554 | 1.012208942 | 0.009291066 | 0.264148364 |
| LINC00942 | 384.4986551 | -1.57861752 | 0.00653157 | 0.226392221 |
| LINC02617 | 2.704063532 | -1.543995022 | 0.012142611 | 0.294093143 |
| AC023796.1 | 1.189537541 | -1.857469925 | 0.012375778 | 0.294093143 |
| AC022509.1 | 11.89376909 | -1.309470409 | 0.030948113 | 0.407459723 |
| AC022509.4 | 1.473148185 | 1.505325431 | 0.003530445 | 0.170957301 |
| AC022079.1 | 13.11935953 | -1.013237422 | 0.002024885 | 0.126430311 |
| OVCH1-AS1 | 2.787691162 | -1.199787237 | 0.005629833 | 0.212779123 |
| AC025154.2 | 17.46269056 | -1.392380047 | 0.01104475 | 0.285017546 |
| AC078864.1 | 2.666136349 | -1.55539302 | 0.016966211 | 0.330272457 |
| AC055716.3 | 0.984617028 | -1.779578446 | 0.038168871 | 0.429888826 |
| HOTAIR | 62.0859048 | -1.242848384 | 0.012223241 | 0.294093143 |
| HOXC-AS3 | 21.15925554 | -1.353061732 | 0.009469555 | 0.266985389 |
| AC020637.1 | 1.334052286 | -1.961042751 | 0.001647566 | 0.114807236 |
| LINC02388 | 2.109954042 | -2.33676057 | 0.001371156 | 0.10587547 |
| LINC02448 | 1.289648422 | -2.082346744 | 0.013042108 | 0.302937419 |
| LINC02384 | 76.4245154 | -1.124695662 | 0.004805764 | 0.194293419 |
| AC090116.1 | 3.46208453 | -1.603003583 | 0.023230447 | 0.374968288 |
| AC090502.2 | 0.385856602 | 1.55304503 | 0.049844042 | NA |
| LNCOG | 17.51784497 | 1.775429411 | 1.19E-07 | 0.000203676 |
| AC027288.3 | 23.14518072 | -1.046971183 | 0.000334037 | 0.050228683 |
| AC027288.1 | 3.534221674 | -1.483739486 | 0.000920141 | 0.089438201 |
| LINC02458 | 3.041692331 | 1.313401261 | 0.000233149 | 0.040782046 |
| CLLU1-AS1 | 10.30674572 | -1.288070847 | 0.005262281 | 0.204086002 |
| AC010205.1 | 0.622528632 | -1.239120214 | 0.026515671 | NA |
| AC079907.2 | 0.818139299 | -1.007533013 | 0.049725409 | 0.459479215 |
| LINC02826 | 1.8074884 | -2.624135112 | 0.002351106 | 0.137054723 |
| LINC00944 | 33.93532619 | 1.100780492 | 0.003004616 | 0.155135942 |
| LINC02418 | 0.830994536 | 1.497942929 | 0.011272686 | 0.285017546 |
| AC138466.1 | 2.736970905 | 1.198370575 | 0.003462932 | 0.169089674 |
| AC148477.3 | 3.431894476 | 1.793242591 | 0.014318966 | 0.310901453 |
| AC148477.2 | 6.555157783 | 1.318816216 | 0.03932257 | 0.430167611 |
| AC148477.4 | 1.324449096 | 2.169668846 | 0.007618329 | 0.244556905 |
| FAM230C | 7.72684197 | -2.506677291 | 0.011464217 | 0.286290596 |
| SPATA13-AS1 | 0.356722554 | 1.241675139 | 0.044485681 | NA |
| LINC01048 | 1.069618799 | 1.221484687 | 0.016258212 | 0.327714191 |
| LINC00332 | 1.396836284 | -1.479351205 | 0.035964763 | 0.427440343 |
| NRAD1 | 5.994394122 | -1.032181359 | 0.04006971 | 0.430912783 |
| LINC00462 | 20.18212936 | -1.493398909 | 0.009990971 | 0.274463505 |
| LINC00348 | 1.063519002 | -2.067679798 | 0.011178395 | 0.285017546 |
| LINC00393 | 27.29540285 | -1.435833548 | 0.014366144 | 0.310901453 |
| GPC5-AS1 | 0.778948137 | -1.981382279 | 0.027120943 | 0.392213166 |
| AL354811.1 | 4.508357224 | 1.159429202 | 0.002366597 | 0.137054723 |
| LINC00391 | 3.495261912 | -1.253938908 | 0.006787182 | 0.228129177 |
| CLDN10-AS1 | 5.58056284 | 2.092792836 | 0.020363653 | 0.355128982 |
| LINC00460 | 54.0607443 | 1.540948395 | 0.003734657 | 0.175877719 |
| AL162574.2 | 13.90756701 | -1.68180786 | 0.001973945 | 0.125764843 |
| AL161431.1 | 719.1150597 | 1.152356852 | 0.029049905 | 0.399553766 |
| LINC00404 | 1.156308851 | -2.2702096 | 0.024928612 | 0.380203024 |
| AL589182.1 | 3.332817467 | -1.267606955 | 0.041636738 | 0.437103821 |
| EGILA | 13.81687078 | -1.112994905 | 0.01010547 | 0.275203224 |
| AC243965.1 | 0.706948662 | -1.155108073 | 0.04552736 | NA |
| AL135999.3 | 4.681677169 | -1.74064095 | 0.001202538 | 0.100067479 |
| LINC02293 | 2.729661109 | 1.483944821 | 0.02808405 | 0.394235341 |
| LINC02300 | 5.061703943 | -1.480453338 | 0.023573911 | 0.375152352 |
| AL133372.2 | 2.81256542 | -1.147029045 | 0.038819195 | 0.430167611 |
| LINC02313 | 3.0730204 | 1.494371711 | 0.000432355 | 0.056984035 |
| AL133304.2 | 0.742869832 | -1.696728927 | 0.041798113 | NA |
| AL162511.1 | 10.47032344 | -1.205738025 | 0.010974765 | 0.285017546 |
| AL121790.2 | 32.63656394 | -1.0620227 | 0.021217282 | 0.355128982 |
| AL121790.1 | 7.200902833 | -1.085924378 | 0.01438796 | 0.310901453 |
| AL161752.1 | 1.897641078 | 1.405544619 | 0.015802383 | 0.324801504 |
| LINC02310 | 4.419735989 | -1.040131755 | 0.045987859 | 0.452539542 |
| AL161757.5 | 2.777140951 | 1.357526619 | 0.03784886 | 0.429888826 |
| AL391261.4 | 0.676723066 | 1.150706752 | 0.012080328 | NA |
| AL133370.1 | 26.77089293 | -4.997608179 | 8.01E-10 | 3.43E-06 |
| AC005993.1 | 36.91773223 | 1.181556966 | 0.001217678 | 0.10035307 |
| LINC01629 | 35.10039843 | 1.758778492 | 0.001750415 | 0.118132342 |
| LINC02308 | 2.919449374 | -1.189025909 | 0.017031901 | 0.330272457 |
| AL049775.3 | 1.090979393 | 1.386486993 | 0.016917217 | 0.330272457 |
| AL357093.1 | 1.609191235 | -1.046206014 | 0.049622633 | 0.459479215 |
| FAM181A-AS1 | 7.186706848 | -1.363177005 | 0.004185362 | 0.182053649 |
| MEG8 | 4.441024976 | -1.199600262 | 0.017152752 | 0.330285661 |
| LINC00524 | 3.791387819 | 1.289970245 | 0.024987371 | 0.380203024 |
| AC055874.1 | 3.965366122 | -2.324470472 | 0.015437279 | 0.321929244 |
| AC087516.2 | 1.566080862 | -1.987263709 | 0.031410203 | 0.410112666 |
| AC090515.4 | 7.070469392 | -1.1178689 | 0.028369328 | 0.39538608 |
| AC073167.1 | 0.519775533 | 1.189349264 | 0.007997461 | NA |
| EWSAT1 | 43.7845258 | -1.216976341 | 0.005417273 | 0.206361969 |
| AC027088.2 | 1.80483241 | -1.962064724 | 0.014386964 | 0.310901453 |
| CT62 | 7.741057385 | 1.61478838 | 4.75E-05 | 0.014808301 |
| AC010931.1 | 15.83044328 | -1.17051348 | 0.001775741 | 0.118905298 |
| AC105020.2 | 1.028937802 | -1.202662178 | 0.014784754 | 0.315199427 |
| AC027807.2 | 4.233336201 | -1.094840502 | 0.02363578 | 0.375152352 |
| AGBL1-AS1 | 2.097575516 | 1.233443114 | 0.046605762 | 0.454445946 |
| LINC00052 | 1.33986026 | -1.817787101 | 0.024117146 | 0.376354148 |
| CRAT37 | 1.402571386 | -1.319540281 | 0.016326445 | 0.327714191 |
| AC091544.2 | 8.335357318 | -1.26772914 | 0.004193309 | 0.182053649 |
| LINC01579 | 56.57848098 | 1.383547713 | 0.002689942 | 0.145680543 |
| LINC02251 | 1.5255895 | -2.253943733 | 0.006169177 | 0.218495946 |
| AC015712.4 | 1.545475651 | -1.379305295 | 0.028183743 | 0.394235341 |
| Z97653.1 | 3.82889888 | -1.131345147 | 0.001073847 | 0.094886019 |
| CEROX1 | 88.28320052 | -1.139624586 | 0.000201322 | 0.040782046 |
| AC009041.1 | 1.645566772 | -1.290535514 | 0.008870036 | 0.262759044 |
| AC087190.2 | 1.360966644 | 1.360003594 | 0.017568128 | 0.336860005 |
| LINC02177 | 1.266146193 | -1.124528684 | 0.045973179 | 0.452539542 |
| AC007601.1 | 2.184244025 | -1.08996723 | 0.011823937 | 0.291215403 |
| AC012317.1 | 0.658085603 | -1.395337931 | 0.048679614 | NA |
| LINC02178 | 11.07282093 | 1.251629061 | 0.02409862 | 0.376354148 |
| AC109462.1 | 5.237112783 | -1.263639005 | 0.00425833 | 0.182237124 |
| AC109462.2 | 5.364932153 | -1.130104817 | 0.012293865 | 0.294093143 |
| AC018552.2 | 1.980692043 | -1.012746227 | 0.02915436 | 0.399553766 |
| AC044802.2 | 1.349604094 | 1.271164597 | 0.001169216 | 0.098392955 |
| AC099521.2 | 5.342489274 | -1.093520782 | 0.009535244 | 0.26783553 |
| LINC01228 | 5.534603822 | -2.961165227 | 0.000213317 | 0.040782046 |
| AC092130.1 | 0.904414877 | 2.041588418 | 0.003223634 | 0.161632946 |
| AC092127.2 | 2.982193347 | -2.419146468 | 3.80E-05 | 0.012578745 |
| AC135012.3 | 1.986445549 | -1.354360445 | 0.014967468 | 0.315199427 |
| AC106745.1 | 0.573796531 | -1.428899927 | 0.030505075 | NA |
| AC010536.3 | 1.58649344 | 1.109754824 | 0.005758934 | 0.21497409 |
| AC137932.2 | 7.104404089 | 1.372021717 | 2.14E-08 | 4.58E-05 |
| AC133919.1 | 0.77482011 | 1.042895448 | 0.018228137 | 0.340638784 |
| AC015921.1 | 4.452486602 | 1.024441555 | 0.033561217 | 0.417710224 |
| AC015908.3 | 4.844005974 | 1.191328203 | 3.82E-05 | 0.012578745 |
| AC104024.2 | 9.262620362 | 1.388098756 | 0.001614556 | 0.114807236 |
| AC069061.2 | 5.796927272 | -2.442149018 | 0.031568318 | 0.410112666 |
| AC011840.1 | 1.227258352 | -2.496290874 | 0.016950239 | 0.330272457 |
| AC079336.3 | 0.544847504 | -1.288199186 | 0.016500278 | NA |
| AC011824.2 | 0.72719536 | -1.113836931 | 0.030315768 | NA |
| LHX1-DT | 8.408030271 | 2.435624594 | 0.000969695 | 0.091332527 |
| FAM215A | 1.165464876 | -1.128719711 | 0.026972108 | 0.391939024 |
| LINC02086 | 51.60700907 | -1.139773051 | 0.009241891 | 0.264040825 |
| AC002401.4 | 19.28100367 | 1.633813524 | 0.000578605 | 0.070845988 |
| AC025048.1 | 1.359704794 | -1.236299373 | 0.014225184 | 0.310901453 |
| AC005544.1 | 0.901110406 | -1.452265173 | 0.020817139 | 0.355128982 |
| ROCR | 1.333893807 | -1.714010616 | 0.043221949 | 0.441851033 |
| AC016168.2 | 2.316602427 | 1.096868719 | 0.033384156 | 0.417710224 |
| AC068594.1 | 1.345438088 | 1.02820466 | 0.007270981 | 0.23786097 |
| AC021683.2 | 49.70932721 | 1.093164311 | 0.047186508 | 0.455214555 |
| AC021683.1 | 27.92551804 | 1.243573678 | 0.026649278 | 0.391499136 |
| AP000894.2 | 11.17073325 | -1.490385073 | 0.003563514 | 0.171589223 |
| AP005328.1 | 4.673809688 | 1.031987717 | 0.024419195 | 0.377792279 |
| AP005264.4 | 1.296208582 | -1.21427472 | 0.042113378 | 0.437186986 |
| LINC01443 | 1.424006017 | 2.039656989 | 0.016750181 | 0.330272457 |
| LINC01444 | 2.011162361 | 4.893126112 | 0.000344517 | 0.050911347 |
| AQP4-AS1 | 18.90924432 | -1.02601997 | 0.024320841 | 0.376951045 |
| AC022601.1 | 0.537651317 | 1.368502683 | 0.016256512 | NA |
| AC120349.1 | 2.805454241 | -1.13261439 | 0.011718835 | 0.289458601 |
| LINC01926 | 1.327555984 | 1.908107965 | 0.000252392 | 0.042416746 |
| AC040896.1 | 0.550400115 | 1.004254793 | 0.021525224 | NA |
| AL136090.1 | 0.602044911 | -1.345882088 | 0.033377997 | NA |
| AL121761.1 | 68.50520797 | -1.224868533 | 0.000928713 | 0.089438201 |
| AL158013.1 | 1.302441206 | 2.610825962 | 0.004572838 | 0.190912452 |
| LINC01747 | 2.208413647 | 1.61308288 | 1.77E-05 | 0.009481206 |
| AL049651.2 | 1.165791906 | -1.870875094 | 0.019934059 | 0.353005834 |
| RIPOR3-AS1 | 1.127295163 | -2.01902796 | 0.00086299 | 0.08698465 |
| GNAS-AS1 | 16.0421966 | -1.062258915 | 0.003328795 | 0.164045804 |
| AL132655.1 | 5.853083586 | -1.048125579 | 0.037217976 | 0.429888826 |
| C20orf197 | 61.1222163 | 1.286787076 | 0.001371097 | 0.10587547 |
| BX640514.1 | 0.449430246 | 1.750467707 | 0.009555249 | NA |
| BX640514.2 | 13.92221752 | 1.503984578 | 5.01E-05 | 0.014808301 |
| AC005256.1 | 10.37823392 | -1.186702439 | 0.041767358 | 0.437103821 |
| AC010636.1 | 0.535829957 | 1.909248903 | 0.007894665 | NA |
| AC005381.1 | 4.846351501 | -3.473645785 | 3.55E-05 | 0.012578745 |
| AC092071.1 | 8.185767913 | -1.625892 | 0.003981298 | 0.182053649 |
| AC005392.2 | 18.02884129 | -1.488378097 | 0.001719091 | 0.117873499 |
| AC006262.3 | 1.268817871 | -1.834078724 | 0.016993772 | 0.330272457 |
| AC007193.2 | 1.113936768 | -1.44534969 | 0.019995374 | 0.353361555 |
| AC011483.1 | 2.147441809 | 1.197648762 | 0.03908497 | 0.430167611 |
| AC011468.3 | 1.698969026 | 1.011884328 | 0.000562704 | 0.069897566 |
| AC022150.3 | 0.961537164 | 1.433511529 | 0.031511489 | 0.410112666 |
| AC010328.1 | 5.300716972 | 2.048363211 | 2.14E-05 | 0.010788529 |
| AC011453.1 | 1.645812789 | 1.190158956 | 0.000450265 | 0.0576003 |
| AC010327.4 | 4.032496584 | 1.480480081 | 0.002730385 | 0.145680543 |
| LINC00278 | 10.31184464 | -1.212810561 | 0.030880385 | 0.407193505 |
| PSLNR | 5.748107415 | -2.036098844 | 0.007843493 | 0.246251201 |
| HORMAD2-AS1 | 4.874501164 | 1.019223936 | 0.001841047 | 0.122322575 |
| AL022316.1 | 13.88910821 | -1.105263394 | 0.002237921 | 0.134134421 |
| LINC01639 | 7.599414223 | -2.750866494 | 0.000148855 | 0.033956824 |
| Z85994.1 | 0.898107726 | 1.936365952 | 0.026236248 | 0.389049965 |
| AP001172.1 | 2.164009385 | -1.499804082 | 0.038887348 | 0.430167611 |
| AF212831.1 | 1.61637818 | -1.044530044 | 0.027714441 | 0.394235341 |
| DSCR4 | 2.376863465 | -3.118550991 | 0.039356874 | 0.430167611 |
| B3GALT5-AS1 | 24.88087325 | -1.143135334 | 0.016090414 | 0.32704612 |
| ERVH48-1 | 16.29451474 | -1.489675563 | 0.000556012 | 0.069897566 |
| FRGCA | 1.368354359 | 1.414804715 | 0.017742582 | 0.338823196 |
| LINC02575 | 134.4147891 | -1.775994553 | 0.001624732 | 0.114807236 |
| TSPEAR-AS1 | 146.3573758 | -1.443975917 | 0.000893163 | 0.08799198 |
| TSPEAR-AS2 | 206.6862083 | -1.40106946 | 0.00116853 | 0.098392955 |
| LINC00163 | 3.397658964 | -1.207989493 | 0.01667917 | 0.330272457 |
| AP001476.1 | 5.91602944 | -1.374972111 | 0.006215857 | 0.219243245 |
| FTCD-AS1 | 0.783691247 | -1.998632336 | 0.005905248 | 0.21497409 |
