# Supplementary material for: A Combined RNA Signature Predicts Recurrence Risk of Stage I-IIIA Lung Squamous Cell Carcinoma
Source: Front Genet. 2021 Jun 14;12:676464. doi: 10.3389/fgene.2021.676464 (PMC8236863; doi:10.3389/fgene.2021.676464)
Supplement: Supplementary file 2 [file Table_2.DOCX]

**Supplementary Table 2**: Differentially expressed microRNAs

| ID | baseMean | log2FoldChange | pvalue | padj |
| --- | --- | --- | --- | --- |
| hsa-mir-122 | 0.710721755 | -1.944824891 | 0.048783125 | NA |
| hsa-mir-1248 | 47.43663408 | 1.110742065 | 0.003795884 | 0.095008758 |
| hsa-mir-1269a | 1589.524031 | -1.2595808 | 0.013707919 | 0.18434344 |
| hsa-mir-1295b | 2.380116458 | -2.079244449 | 0.001343425 | 0.068380343 |
| hsa-mir-1304 | 8.232239636 | 2.118415867 | 2.12E-07 | 0.000107666 |
| hsa-mir-137 | 9.915067918 | -1.866288058 | 0.001119951 | 0.063339454 |
| hsa-mir-147b | 6.768237952 | 1.087164709 | 0.000907142 | 0.063339454 |
| hsa-mir-184 | 30.85676015 | -1.609245265 | 0.000517798 | 0.05271179 |
| hsa-mir-23c | 1.074071468 | -1.379456961 | 0.009829199 | NA |
| hsa-mir-3617 | 0.424106314 | 1.424059862 | 0.024340699 | NA |
| hsa-mir-431 | 57.18863124 | -1.118069522 | 0.000910485 | 0.063339454 |
| hsa-mir-449a | 11.83407506 | -2.109955099 | 0.000377866 | 0.048083416 |
| hsa-mir-449b | 2.13518945 | -2.2229971 | 0.006700333 | 0.136418773 |
| hsa-mir-449c | 1.242956642 | -2.317577285 | 0.003265644 | NA |
| hsa-mir-4732 | 1.489730688 | -1.105817597 | 0.019719606 | NA |
| hsa-mir-506 | 3.517652333 | -1.385557681 | 0.018791889 | 0.217387994 |
| hsa-mir-509-1 | 21.90411501 | -1.388897083 | 0.003571452 | 0.095008758 |
| hsa-mir-509-2 | 21.38481414 | -1.283900792 | 0.005094824 | 0.113925899 |
| hsa-mir-509-3 | 24.18189349 | -1.065630497 | 0.018541611 | 0.217387994 |
| hsa-mir-512-2 | 0.833950009 | 1.324054536 | 0.016205842 | NA |
| hsa-mir-514a-1 | 28.38233943 | -1.283782355 | 0.005147929 | 0.113925899 |
| hsa-mir-514a-2 | 27.6361258 | -1.250812685 | 0.007278608 | 0.142492756 |
| hsa-mir-514a-3 | 28.24062492 | -1.370135627 | 0.003469323 | 0.095008758 |
| hsa-mir-516b-1 | 0.520885821 | 1.739021976 | 0.024461865 | NA |
| hsa-mir-518a-2 | 0.303948816 | 1.469444619 | 0.018638519 | NA |
| hsa-mir-548f-1 | 3.719456662 | -1.678146859 | 0.00574406 | 0.121821944 |
| hsa-mir-548x | 0.617873251 | 1.755978942 | 0.047888857 | NA |
| hsa-mir-675 | 144.5995673 | 1.672914463 | 8.86E-05 | 0.022555458 |
| hsa-mir-6814 | 1.073155692 | -1.252587194 | 0.005473486 | NA |
| hsa-mir-6825 | 0.376781526 | 1.187197379 | 0.014599645 | NA |
| hsa-mir-6844 | 0.490160331 | 1.386924519 | 0.019760455 | NA |
| hsa-mir-7641-1 | 4.628552167 | -1.093235913 | 0.027291467 | 0.254005416 |
| hsa-mir-888 | 1.0031108 | -2.544311926 | 0.027212303 | NA |
| hsa-mir-891a | 109.8314417 | -2.00615744 | 0.000166133 | 0.028187261 |
| hsa-mir-892a | 2.348122605 | -2.321403082 | 0.009718271 | 0.154581241 |
| hsa-mir-934 | 8.836406153 | -1.143875211 | 0.008858246 | 0.15439257 |
