# Supplementary material for: A Combined RNA Signature Predicts Recurrence Risk of Stage I-IIIA Lung Squamous Cell Carcinoma
Source: Front Genet. 2021 Jun 14;12:676464. doi: 10.3389/fgene.2021.676464 (PMC8236863; doi:10.3389/fgene.2021.676464)
Supplement: Supplementary file 3 [file Table_3.DOCX]

**Supplementary Table 3**: Differentially expressed mRNAs

| ID | baseMean | log2FoldChange | pvalue | padj |
| --- | --- | --- | --- | --- |
| CA6 | 6.482113436 | -1.205742211 | 0.0155306 | 0.23175166 |
| ANGPTL7 | 5.107270224 | -1.037983241 | 0.0158191 | 0.23340078 |
| DISP3 | 54.29563404 | 2.004278815 | 3.12E-06 | 0.00109289 |
| C1orf158 | 9.514460059 | -1.309448117 | 0.0459169 | 0.38135812 |
| SPATA21 | 7.363115515 | -2.182290688 | 7.95E-05 | 0.01016911 |
| PADI1 | 845.5364225 | -2.213627383 | 4.21E-05 | 0.00614917 |
| ACTL8 | 38.94031997 | -2.478063948 | 7.17E-06 | 0.00208943 |
| CDA | 267.8964045 | 2.211851369 | 1.92E-10 | 3.96E-07 |
| HTR1D | 48.59956769 | 1.135685196 | 0.0029148 | 0.09725138 |
| TEKT2 | 85.10406324 | -1.788018316 | 1.25E-05 | 0.00312175 |
| GRIK3 | 22.92448186 | -2.198087979 | 9.95E-06 | 0.00276957 |
| COL9A2 | 1969.764521 | -1.151223456 | 0.0008376 | 0.04456299 |
| CLDN19 | 50.8592829 | 3.090097529 | 1.38E-05 | 0.00328336 |
| CFAP57 | 167.298273 | -2.106015844 | 1.81E-07 | 0.00012358 |
| TCTEX1D4 | 9.757369162 | -1.708284403 | 6.65E-05 | 0.00875327 |
| CYP4B1 | 893.8435099 | -2.324334801 | 5.82E-07 | 0.00029983 |
| GLIS1 | 48.22475659 | -1.015813597 | 0.0021525 | 0.08301556 |
| LDLRAD1 | 69.54006575 | -1.258121564 | 0.0108116 | 0.1962481 |
| L1TD1 | 20.76075933 | 1.276336238 | 0.0009199 | 0.04793376 |
| ERICH3 | 88.08923734 | -1.233637198 | 0.0117138 | 0.20442324 |
| AK5 | 19.9992478 | -1.171301635 | 0.0005713 | 0.03558143 |
| PTGFR | 64.87453538 | -1.102512707 | 0.0006022 | 0.0362696 |
| WDR63 | 36.35366551 | -1.101676877 | 0.0022275 | 0.08381676 |
| OLFM3 | 4.379161561 | -2.074064989 | 0.0050629 | 0.12891356 |
| SYCP1 | 1.501297043 | -1.527744347 | 0.0374926 | 0.34915882 |
| HMGCS2 | 28.18950427 | -1.520925938 | 0.0209334 | 0.26486023 |
| TCHH | 648.436378 | -1.528900199 | 0.0011295 | 0.05456815 |
| RPTN | 166.4699758 | -2.639007582 | 1.88E-05 | 0.0037196 |
| FLG2 | 12.0158778 | -1.079593253 | 0.0206118 | 0.26329466 |
| CRNN | 62.70629571 | -2.812058925 | 0.0002384 | 0.0207644 |
| LCE3E | 29.9507755 | -1.234663538 | 0.0495604 | 0.38877518 |
| C1orf68 | 11.86097807 | 1.286126774 | 0.0482556 | 0.38647299 |
| SMCP | 6.712208609 | -1.710497034 | 0.0134656 | 0.21713788 |
| SPRR4 | 76.09532747 | -1.729440879 | 0.0059268 | 0.14186746 |
| LORICRIN | 15.07571591 | -2.236168722 | 2.16E-05 | 0.00417787 |
| S100A7A | 551.6669804 | -1.541350604 | 0.0129689 | 0.21367627 |
| S100A7 | 3222.660532 | -1.327580755 | 0.0213925 | 0.26780001 |
| PKLR | 4.153883259 | 1.166893046 | 0.010035 | 0.19073725 |
| INSRR | 14.80100447 | -1.059704304 | 0.0055415 | 0.13473169 |
| LRRC71 | 26.48059619 | -1.036002595 | 0.0135241 | 0.2172282 |
| ATP1A2 | 45.9079297 | -1.228046754 | 0.0032972 | 0.10330997 |
| ITLN2 | 52.34460663 | -1.364093428 | 0.00084 | 0.04456299 |
| APOA2 | 7.901578834 | 1.954568875 | 0.0012144 | 0.05747138 |
| MAEL | 21.19258128 | -1.389498549 | 0.0096988 | 0.18702865 |
| CCDC181 | 55.5816136 | 1.088841349 | 0.000679 | 0.03875618 |
| MROH9 | 6.775519972 | -1.168432249 | 0.0092183 | 0.18196294 |
| FMO3 | 960.8239381 | -2.080373593 | 3.24E-08 | 3.16E-05 |
| SLC9C2 | 7.283710436 | -1.072098924 | 0.0103885 | 0.19329741 |
| SERPINC1 | 7.225320758 | 1.167212628 | 0.0028205 | 0.0959407 |
| TNR | 17.24761547 | -1.015632358 | 0.0155975 | 0.23215273 |
| ASTN1 | 44.88137302 | -1.54649288 | 0.0018184 | 0.07423538 |
| FAM163A | 13.18276027 | -1.194184774 | 0.002901 | 0.09713998 |
| B3GALT2 | 36.63749981 | -1.588329174 | 2.09E-05 | 0.00407688 |
| F13B | 0.545355126 | 1.568024005 | 0.0177074 | 0.24477693 |
| CRB1 | 3.923849914 | -1.139280802 | 0.0060475 | 0.14346265 |
| SYT2 | 113.5463292 | -1.070480925 | 0.0003497 | 0.02680843 |
| MYOG | 0.695921861 | -1.617370896 | 0.0398946 | 0.35856857 |
| PRELP | 2154.971859 | -1.161627287 | 0.0001131 | 0.01298195 |
| CNTN2 | 23.26175664 | -1.750773429 | 0.0001344 | 0.01475591 |
| IL20 | 17.40845294 | 2.881856828 | 2.22E-07 | 0.00013704 |
| FCAMR | 14.64714753 | -1.052018278 | 0.021411 | 0.26782702 |
| CR2 | 308.1084058 | -1.043022741 | 0.0237373 | 0.28191883 |
| HHIPL2 | 93.48821527 | 1.719830644 | 0.0002156 | 0.0198969 |
| LEFTY1 | 8.601221272 | 1.387441668 | 6.91E-05 | 0.00896168 |
| LEFTY2 | 10.75083836 | -1.494766942 | 0.0002966 | 0.02413071 |
| ZP4 | 5.132166354 | -4.069790706 | 3.01E-05 | 0.00503123 |
| TRIM58 | 35.89767405 | 1.746586729 | 0.0009641 | 0.04914713 |
| OR2W3 | 5.714771283 | 1.903338736 | 0.0010553 | 0.05220473 |
| OR2T8 | 0.742184888 | 2.300260974 | 0.0197666 | 0.25724994 |
| KCNF1 | 25.54978189 | 1.215846826 | 0.0016702 | 0.07005307 |
| MYCN | 174.763621 | -1.146245045 | 0.008412 | 0.17276849 |
| MSGN1 | 13.5095122 | -1.246015205 | 0.0144056 | 0.22269821 |
| DRC1 | 106.1082309 | -1.203597225 | 0.0103117 | 0.19310546 |
| TOGARAM2 | 101.1718865 | -1.013151325 | 0.0007446 | 0.04088591 |
| ALK | 59.69113871 | -1.090895144 | 0.0031295 | 0.10096443 |
| CAPN14 | 351.1304555 | -1.546032618 | 0.0002008 | 0.01929806 |
| VIT | 46.16386138 | -1.904665833 | 6.00E-06 | 0.00179558 |
| CDKL4 | 7.150367694 | -1.14081947 | 0.001313 | 0.06080134 |
| NRXN1 | 144.7841216 | -2.340802659 | 1.81E-05 | 0.00369467 |
| C2orf73 | 7.842672925 | -1.280563176 | 0.0033135 | 0.10330997 |
| GKN2 | 31.68827412 | -1.780928441 | 0.0020274 | 0.07968388 |
| ASPRV1 | 108.3813614 | -1.046015409 | 0.0003489 | 0.02680843 |
| LRRTM4 | 22.54793359 | -1.429261248 | 0.006435 | 0.14921921 |
| CTNNA2 | 2.773498532 | -1.692327564 | 0.0022937 | 0.08561364 |
| DNAH6 | 109.3879822 | -1.219294208 | 0.0019462 | 0.07777023 |
| TEX37 | 0.619567884 | 1.87393641 | 0.0009423 | 0.04855809 |
| MAL | 201.8545659 | -1.250798555 | 0.0004685 | 0.03267077 |
| CNGA3 | 4.006884742 | -1.90324729 | 0.0001756 | 0.01752928 |
| ECRG4 | 55.25702329 | -1.209081814 | 0.0127528 | 0.21251532 |
| RGPD3 | 9.423311345 | -1.045426413 | 0.0018144 | 0.07423538 |
| SLC5A7 | 148.6842733 | -2.158902298 | 0.0027585 | 0.0959407 |
| IL1A | 731.567662 | 1.095611184 | 0.0129934 | 0.21367627 |
| POTEF | 37.00461149 | -1.148384884 | 0.0051892 | 0.13061778 |
| THSD7B | 168.3982173 | -2.003162999 | 1.81E-05 | 0.00369467 |
| KCNJ3 | 3.49102071 | -1.430857461 | 0.0130614 | 0.21404713 |
| DAPL1 | 2681.832969 | -1.108874858 | 0.0095863 | 0.1856311 |
| DHRS9 | 657.4073225 | -1.269145687 | 0.000278 | 0.02333835 |
| CHRNA1 | 42.36280037 | -1.394640818 | 3.45E-05 | 0.0053773 |
| FRZB | 384.0752947 | -1.22510242 | 2.39E-05 | 0.0045485 |
| TMEFF2 | 7.391241198 | -1.017065114 | 0.0189783 | 0.25219666 |
| IHH | 8.569645185 | -1.422381838 | 0.001203 | 0.05722351 |
| DES | 306.2688832 | -1.613387453 | 0.0001436 | 0.0156611 |
| SCG2 | 229.1275284 | -1.560465883 | 1.19E-05 | 0.00303662 |
| ALPP | 15.3828679 | -1.404806768 | 0.0032226 | 0.10250853 |
| ECEL1 | 134.7247282 | -1.440025476 | 0.0005843 | 0.03562355 |
| EFHD1 | 274.5927787 | 1.084149304 | 0.0016463 | 0.06940846 |
| UGT1A8 | 33.29921951 | -2.100936129 | 0.0023041 | 0.08565852 |
| UGT1A10 | 404.7233571 | -2.028295775 | 0.0103859 | 0.19329741 |
| KIF1A | 1948.018219 | 1.187645021 | 0.0305839 | 0.31856411 |
| CROCC2 | 34.46273548 | -1.072075667 | 0.0174617 | 0.2435577 |
| CNTN6 | 15.3079539 | -1.076838323 | 0.026597 | 0.29777845 |
| IL5RA | 55.06404175 | -1.140104892 | 0.0043898 | 0.11970513 |
| LHFPL4 | 20.26276682 | 3.744365957 | 1.05E-13 | 6.51E-10 |
| DAZL | 8.177086798 | -1.21680179 | 0.0088751 | 0.17779928 |
| SCN10A | 1.037801914 | -1.369348623 | 0.0414853 | 0.36440076 |
| MOBP | 43.26661449 | -1.273580082 | 0.0071201 | 0.15990967 |
| CCK | 7.386219014 | 1.230263874 | 0.0361137 | 0.34373451 |
| HHATL | 4.080096224 | -1.15017929 | 0.03333 | 0.33293288 |
| KRBOX1 | 41.03080456 | -1.032098695 | 0.0408839 | 0.36167699 |
| CLEC3B | 185.6866915 | -1.110049234 | 0.0001546 | 0.01625562 |
| XCR1 | 94.06220801 | -1.102031493 | 0.0005478 | 0.03550231 |
| SPINK8 | 3.104932575 | -2.166343417 | 0.0004307 | 0.03061319 |
| CDHR4 | 42.56426544 | -1.116979284 | 0.0194891 | 0.25550661 |
| GNAT1 | 2.752619466 | -1.036123086 | 0.0136318 | 0.21748229 |
| TNNC1 | 67.19280724 | -1.279993997 | 0.0001177 | 0.01315637 |
| DNAH12 | 43.64512662 | -1.275028725 | 0.0013829 | 0.0630146 |
| FAM107A | 314.3877856 | -1.350521723 | 0.0001694 | 0.01726848 |
| CADPS | 26.57620301 | -1.487268161 | 1.88E-05 | 0.0037196 |
| CADM2 | 17.11254739 | -1.455728781 | 0.0022213 | 0.08375641 |
| EPHA3 | 173.5860881 | -1.230211083 | 0.0001615 | 0.0166198 |
| HHLA2 | 44.18512538 | -1.052432252 | 0.0045706 | 0.12264838 |
| MORC1 | 3.554842517 | -3.124336446 | 0.0009643 | 0.04914713 |
| LSAMP | 751.5195172 | -1.271445814 | 8.11E-05 | 0.01023502 |
| CASR | 9.904356806 | -2.281042698 | 2.73E-05 | 0.00477189 |
| MUC13 | 99.60862711 | -1.025595807 | 0.0445141 | 0.37530694 |
| KBTBD12 | 44.90070368 | -2.051920944 | 3.45E-05 | 0.0053773 |
| GP9 | 0.987495643 | -1.398055958 | 0.0371459 | 0.34730293 |
| COL6A5 | 168.0565269 | -1.232428707 | 0.0006054 | 0.0362696 |
| COL6A6 | 312.365235 | -1.32048024 | 0.0004108 | 0.02965587 |
| CLDN18 | 386.0717754 | -1.628522845 | 0.0004028 | 0.0294209 |
| CLRN1 | 0.5174317 | 1.340900525 | 0.0254932 | 0.29247059 |
| AADACL2 | 61.10100025 | -1.750732197 | 0.0006653 | 0.03849371 |
| VEPH1 | 139.9007031 | -1.116064726 | 0.0022059 | 0.08368366 |
| SLITRK3 | 25.19273629 | -1.10014718 | 0.0491275 | 0.38877518 |
| ZBBX | 31.70730017 | -1.19101545 | 0.0191576 | 0.25349013 |
| SERPINI2 | 17.32471624 | -1.113786445 | 0.012788 | 0.21257112 |
| SAMD7 | 0.782700882 | -1.487131797 | 0.0367392 | 0.34543768 |
| CLDN11 | 300.2912327 | -1.342076357 | 0.0002211 | 0.02007162 |
| SLC7A14 | 3.856318542 | -1.61678475 | 0.0040865 | 0.11451588 |
| TMEM212 | 7.932128573 | -1.410462582 | 0.0214394 | 0.26782702 |
| HTR3E | 3.310353673 | -1.103438784 | 0.0410796 | 0.36271633 |
| ADIPOQ | 1.436729022 | 1.834286458 | 0.0464459 | 0.38243138 |
| SST | 1.861239397 | -1.863576486 | 0.0268378 | 0.29891782 |
| CLDN16 | 296.0295522 | -1.125529596 | 0.0013755 | 0.06284848 |
| GMNC | 29.48676982 | -3.851278482 | 1.06E-08 | 1.16E-05 |
| OSTN | 3.166146122 | -2.722703987 | 3.23E-05 | 0.00520643 |
| ZNF732 | 14.87567232 | 1.329023238 | 0.0042493 | 0.1180083 |
| BST1 | 254.73091 | 1.065724434 | 0.0001518 | 0.01618376 |
| FGFBP2 | 455.6053265 | -2.457342286 | 1.63E-05 | 0.00356412 |
| DTHD1 | 77.97691821 | -1.245154165 | 0.0008193 | 0.04392897 |
| TLR10 | 129.9922371 | -1.052503145 | 0.0016116 | 0.06872854 |
| BEND4 | 42.18621973 | 1.307315114 | 0.0046143 | 0.12352151 |
| GABRA2 | 35.69145966 | 1.879463435 | 0.006318 | 0.14687375 |
| GABRA4 | 2.607508715 | -2.437603748 | 0.0288518 | 0.30867068 |
| PDCL2 | 3.033454447 | 1.682109222 | 0.0073165 | 0.1617678 |
| EPHA5 | 14.8053402 | -1.334771877 | 0.0265672 | 0.29777845 |
| TMPRSS11B | 41.94251074 | -2.221038329 | 0.0016359 | 0.06912933 |
| UGT2B17 | 45.29791693 | -2.022113222 | 1.66E-05 | 0.0035852 |
| UGT2B15 | 7.423089757 | -2.126204671 | 0.0001232 | 0.01368672 |
| UGT2A3 | 2.416574171 | -2.170291804 | 0.0094304 | 0.18412537 |
| UGT2B28 | 7.3914745 | -1.646461994 | 0.0071407 | 0.1601782 |
| UGT2A1 | 57.0520631 | -2.656894795 | 2.96E-05 | 0.00499512 |
| SULT1B1 | 90.86291845 | -1.770819625 | 6.82E-07 | 0.00032244 |
| SULT1E1 | 231.4225772 | -2.838127486 | 9.59E-09 | 1.11E-05 |
| CSN1S1 | 2.41103429 | -3.336857409 | 0.0028343 | 0.09594756 |
| STATH | 20.80983701 | -4.386959786 | 9.56E-09 | 1.11E-05 |
| HTN3 | 1.117043021 | -2.315061469 | 0.0119947 | 0.20672709 |
| HTN1 | 1.301571922 | -2.42825088 | 0.0046293 | 0.12362411 |
| PRR27 | 2.042640898 | -3.645720377 | 0.0094241 | 0.18412537 |
| ODAM | 123.9482529 | -4.768466689 | 5.99E-13 | 2.78E-09 |
| FDCSP | 2332.981734 | -5.225185887 | 7.29E-16 | 6.76E-12 |
| CSN3 | 33.38267219 | -7.948091289 | 5.05E-10 | 9.37E-07 |
| MUC7 | 1.754138815 | -3.095612557 | 0.0175999 | 0.2440599 |
| AMTN | 790.2760972 | -3.84767067 | 7.24E-11 | 1.93E-07 |
| SLC4A4 | 247.8158965 | -1.059973438 | 0.0048357 | 0.12669483 |
| GC | 1.815913474 | -3.143860976 | 0.0162609 | 0.2367259 |
| CXCL6 | 895.4109211 | -1.761137412 | 0.0001013 | 0.01220729 |
| PF4V1 | 9.858094166 | -1.998069105 | 0.0002147 | 0.0198969 |
| CXCL1 | 3753.003693 | -1.085182838 | 0.0059676 | 0.14247857 |
| EREG | 264.2815346 | 2.180640679 | 2.67E-05 | 0.00475946 |
| AREG | 1145.784027 | 1.225285293 | 0.000618 | 0.03686499 |
| ODAPH | 17.85238467 | 1.211033892 | 0.0061378 | 0.14467884 |
| CXCL11 | 527.4181989 | 1.384570283 | 0.0010213 | 0.05093083 |
| FGF5 | 10.68556259 | 1.845397506 | 0.0003718 | 0.02783961 |
| BMP3 | 549.1264366 | -1.393738669 | 0.0028054 | 0.0959407 |
| PLAC8 | 943.4481497 | -1.007469907 | 0.0078869 | 0.16726327 |
| HSD17B13 | 43.3455072 | -1.612538405 | 6.87E-05 | 0.00896168 |
| MEPE | 3.790401627 | 1.447638345 | 0.0002057 | 0.019366 |
| ADH1B | 547.4822039 | -1.552093138 | 0.0011349 | 0.0546857 |
| ADH1C | 1270.562399 | -1.333930653 | 0.0262414 | 0.2961092 |
| COL25A1 | 15.93036938 | -1.394967307 | 0.0006941 | 0.03910228 |
| PRSS12 | 1425.906626 | -1.037063507 | 0.0010388 | 0.05153474 |
| SYNPO2 | 1338.855864 | -1.929300158 | 1.21E-07 | 9.46E-05 |
| MYOZ2 | 4.674324915 | -1.180843577 | 0.0046716 | 0.12449042 |
| NDNF | 460.9925562 | -1.314520182 | 0.0006944 | 0.03910228 |
| CLGN | 272.2805446 | 1.230866316 | 0.000565 | 0.03558143 |
| RNF150 | 458.2847793 | -1.356909106 | 0.0001758 | 0.01752928 |
| C4orf51 | 7.58417136 | -1.204282785 | 0.0116446 | 0.20369956 |
| GRIA2 | 17.67447248 | -1.926274628 | 0.0001718 | 0.01736539 |
| RXFP1 | 55.49226552 | -1.157821783 | 0.0003458 | 0.02672662 |
| NPY5R | 2.168466878 | -1.924530735 | 0.0127273 | 0.21251532 |
| ANXA10 | 109.9223151 | 1.073830146 | 0.0480844 | 0.38621259 |
| SCRG1 | 9.917160188 | -1.205506022 | 0.0017344 | 0.07162355 |
| GPM6A | 30.39951327 | -1.496182097 | 0.0003181 | 0.02510852 |
| VEGFC | 333.5592055 | 1.17646044 | 1.53E-05 | 0.00342459 |
| ENPP6 | 63.28918354 | -2.223549866 | 1.55E-06 | 0.00062409 |
| F11 | 13.0295036 | -1.063253331 | 0.0330493 | 0.33176321 |
| MTNR1A | 2.720335135 | -1.001086266 | 0.0326723 | 0.32922578 |
| ZFP42 | 53.26653123 | 2.917339067 | 0.0001033 | 0.01236787 |
| UBE2QL1 | 160.2739475 | -1.121814858 | 0.0040107 | 0.11352512 |
| C5orf49 | 62.79794659 | -1.210875237 | 0.0034812 | 0.1053491 |
| ANKRD33B | 608.5118659 | -1.289318201 | 0.0007445 | 0.04088591 |
| DNAH5 | 534.7729041 | -1.342184915 | 0.0002055 | 0.019366 |
| CDH18 | 28.66780371 | 2.308568694 | 0.0027713 | 0.0959407 |
| CDH12 | 45.92314214 | -2.376533354 | 0.0002402 | 0.02082566 |
| CDH6 | 401.1033171 | -1.61963661 | 5.19E-06 | 0.00157805 |
| SLC45A2 | 10.52182929 | -1.620536092 | 1.80E-05 | 0.00369467 |
| PRLR | 411.404658 | -1.143329868 | 0.0099493 | 0.1899464 |
| CAPSL | 31.34904209 | -1.089321952 | 0.0280393 | 0.30454166 |
| UGT3A1 | 60.44687466 | -2.183088837 | 0.0146645 | 0.22519974 |
| UGT3A2 | 52.87483437 | -2.381942125 | 4.30E-06 | 0.00139866 |
| MROH2B | 1.447077252 | -1.298435287 | 0.0490855 | 0.38877518 |
| HCN1 | 2.360936538 | -2.183507238 | 0.0013859 | 0.0630146 |
| HSPB3 | 94.44084504 | -1.942166623 | 2.40E-05 | 0.0045485 |
| CDC20B | 19.98188322 | -2.109800636 | 1.56E-05 | 0.00343468 |
| DDX4 | 3.237898752 | -1.209894522 | 0.0152463 | 0.22920143 |
| BHMT2 | 53.28195337 | 1.162145144 | 0.0005969 | 0.03607118 |
| SPZ1 | 2.015658649 | 1.495705804 | 0.0179632 | 0.24500133 |
| ACOT12 | 3.084004937 | -1.662764232 | 0.004975 | 0.12836018 |
| LIX1 | 9.492383412 | -2.151288711 | 0.0001177 | 0.01315637 |
| TRIM36 | 79.95866133 | 1.221114807 | 2.36E-06 | 0.00085694 |
| ZNF474 | 27.1273304 | -1.105641801 | 0.0010188 | 0.05093083 |
| SLC27A6 | 44.46555323 | -1.999344257 | 0.0002055 | 0.019366 |
| CSF2 | 32.42855223 | 1.487308171 | 0.0002039 | 0.019366 |
| TGFBI | 14441.9547 | 1.365977673 | 1.58E-06 | 0.0006242 |
| SMIM32 | 5.064653474 | -1.479562874 | 0.0050458 | 0.12888686 |
| PCDHA1 | 19.98269624 | 1.256635025 | 0.0204821 | 0.26313278 |
| PCDHA3 | 23.17305781 | 1.102066588 | 0.0023948 | 0.08739101 |
| PCDHA6 | 21.705183 | 1.300546293 | 0.0042007 | 0.11718262 |
| PCDHA7 | 12.62548265 | 1.661703416 | 4.77E-07 | 0.00026812 |
| PCDHA10 | 49.63307003 | 1.194767708 | 0.0008408 | 0.04456299 |
| PCDHB6 | 210.3453429 | 1.015190041 | 0.0162458 | 0.2367259 |
| PCDHGA3 | 31.09714536 | 2.397314742 | 3.99E-11 | 1.48E-07 |
| PCDHGB2 | 141.861008 | -1.16794996 | 0.000526 | 0.0347239 |
| PCDHGC5 | 44.69976679 | 1.352654995 | 1.74E-05 | 0.00369467 |
| HMHB1 | 0.515343899 | 1.735510055 | 0.0049488 | 0.12822044 |
| NMUR2 | 26.43697306 | -1.103495903 | 0.0294445 | 0.31170659 |
| GRIA1 | 21.0038774 | -1.895436932 | 1.39E-06 | 0.00058476 |
| HAND1 | 4.948396686 | -1.508689574 | 0.0400453 | 0.3592264 |
| GABRA1 | 2.190552579 | -2.839962955 | 0.00069 | 0.03910228 |
| GABRP | 960.0244221 | -2.049805533 | 5.04E-05 | 0.00718809 |
| COL23A1 | 216.8803602 | 1.0798901 | 0.0011154 | 0.05425033 |
| NRSN1 | 2.990548418 | -1.412614565 | 0.0294091 | 0.31166796 |
| SLC17A3 | 1.200741782 | -1.633986462 | 0.0038916 | 0.11245618 |
| H3C1 | 4.748045308 | -1.141569073 | 0.0054526 | 0.13397531 |
| MUCL3 | 44.02735385 | -1.657737305 | 0.0014525 | 0.0644635 |
| C6orf15 | 201.9060076 | -1.429760146 | 0.0222547 | 0.27333955 |
| AGER | 1039.584878 | -1.198851116 | 0.0003163 | 0.02510852 |
| SPDEF | 131.6925327 | -1.382592167 | 0.000586 | 0.03562355 |
| TCP11 | 8.408133203 | -1.023743916 | 0.0250691 | 0.28975485 |
| CLPSL1 | 8.755176379 | -1.809194769 | 0.0039573 | 0.11329011 |
| BNIP5 | 22.5088414 | -1.097550986 | 0.0123936 | 0.20996695 |
| PI16 | 33.81565367 | -1.235771949 | 0.0039879 | 0.11337208 |
| TSPO2 | 4.482899855 | 1.292968291 | 0.0002634 | 0.02251991 |
| TCTE1 | 27.08751455 | -1.425142213 | 0.0010647 | 0.05252805 |
| CLIC5 | 432.4439895 | -1.497488386 | 4.05E-05 | 0.00610673 |
| GLYATL3 | 2.047505323 | -2.172783202 | 0.044935 | 0.3781866 |
| CRISP2 | 12.05095801 | -1.647729168 | 0.0100486 | 0.19080059 |
| CRISP3 | 24.55783584 | -1.539998655 | 0.0104862 | 0.19415441 |
| TFAP2D | 4.581217399 | -2.464151685 | 0.0043943 | 0.11970513 |
| GSTA2 | 29.40086277 | -1.821109242 | 0.0003034 | 0.02439081 |
| GSTA3 | 12.01494511 | -1.909496906 | 9.46E-05 | 0.01162415 |
| COL19A1 | 81.64469533 | -1.993103803 | 0.0002335 | 0.02053042 |
| KHDC1L | 43.89734671 | 1.519186648 | 0.006207 | 0.14557043 |
| NT5E | 1030.250282 | 1.582463106 | 4.02E-06 | 0.00137941 |
| HTR1E | 2.821486012 | -2.198322466 | 0.0110155 | 0.19839709 |
| TRAPPC3L | 14.63370784 | -1.190186413 | 0.0098962 | 0.18965282 |
| FABP7 | 71.80637508 | -2.844310764 | 6.95E-07 | 0.00032244 |
| CLVS2 | 4.645385281 | -2.333993008 | 0.0026787 | 0.09397146 |
| SMLR1 | 0.927793792 | -1.466497682 | 0.0030853 | 0.10084093 |
| ENPP3 | 45.47701927 | -1.596033427 | 2.80E-05 | 0.00484876 |
| MOXD1 | 1980.54874 | -1.170246221 | 0.0004789 | 0.03327141 |
| TCF21 | 106.5414658 | -1.029494619 | 0.0015178 | 0.06609392 |
| MYB | 648.2393386 | 1.223556863 | 0.0001466 | 0.0157155 |
| ADGB | 8.559462624 | -1.338245539 | 0.0284419 | 0.30729557 |
| RAET1E | 83.37565583 | 1.268296116 | 0.0022452 | 0.08431155 |
| OPRM1 | 0.531780027 | 1.397841117 | 0.0352882 | 0.3405511 |
| PNLDC1 | 225.1567575 | -3.253046292 | 2.24E-09 | 3.19E-06 |
| PLG | 3.548357383 | -1.313742656 | 0.0142666 | 0.22184337 |
| TBXT | 3.135337328 | 2.460363798 | 0.0001177 | 0.01315637 |
| UNC93A | 18.59283857 | -1.669222884 | 0.0035512 | 0.10645727 |
| KIF25 | 16.02140767 | -1.203758932 | 0.0009229 | 0.04795679 |
| FRMD1 | 6.337078842 | -1.60253623 | 0.0029903 | 0.09910563 |
| GRIFIN | 1.053332873 | -1.548241824 | 0.0032197 | 0.10250853 |
| AMZ1 | 67.67308459 | 1.431284926 | 4.20E-06 | 0.0013926 |
| NPY | 12.13156897 | -2.84570892 | 0.0007671 | 0.04185342 |
| INMT | 318.6780615 | -1.353064232 | 1.44E-05 | 0.00330637 |
| AC004691.2 | 1.722753788 | -1.238603884 | 0.0035508 | 0.10645727 |
| GHRHR | 2.397184056 | -1.444034008 | 0.0063918 | 0.14840255 |
| MYL7 | 4.037234898 | -1.64289042 | 0.0039027 | 0.11245618 |
| C7orf57 | 32.94782512 | -1.260818137 | 0.0012562 | 0.05884761 |
| DDC | 58.4176943 | -1.924841147 | 2.71E-05 | 0.00477189 |
| SEPTIN14 | 1.119440053 | 1.850785408 | 0.0006577 | 0.03824621 |
| NUPR2 | 44.54645741 | -1.083178386 | 0.0223804 | 0.27425407 |
| CALN1 | 78.18690607 | -1.371213246 | 0.0380154 | 0.35190771 |
| SEMA3E | 120.4631512 | -1.190065807 | 0.0112974 | 0.19997828 |
| STEAP4 | 1446.880556 | -1.14339941 | 0.0020108 | 0.07953596 |
| HEPACAM2 | 18.57947005 | -1.960783374 | 0.0001973 | 0.019067 |
| AZGP1 | 555.4152216 | 2.624847477 | 7.12E-08 | 6.61E-05 |
| VGF | 31.99194436 | 1.660794608 | 1.52E-07 | 0.00010813 |
| COL26A1 | 162.9633031 | -1.774179888 | 1.80E-05 | 0.00369467 |
| LMOD2 | 1.485485317 | -1.041490174 | 0.0270536 | 0.29944571 |
| LEP | 29.52160043 | -2.682972865 | 7.53E-09 | 9.97E-06 |
| FAM71F1 | 43.00635335 | -1.009203488 | 0.0383272 | 0.35285786 |
| FAM180A | 40.41898774 | -1.009784148 | 0.0007559 | 0.04136544 |
| MGAM2 | 13.59494487 | -1.147667951 | 0.0183952 | 0.24782108 |
| KEL | 57.16431757 | -1.169189571 | 0.0008023 | 0.04332909 |
| PIP | 16.75397952 | -2.011319924 | 0.0035593 | 0.10645727 |
| AOC1 | 103.3742084 | -1.066000913 | 0.0015961 | 0.06853828 |
| SHH | 235.4047749 | -1.335482707 | 0.0020966 | 0.08170853 |
| VIPR2 | 17.62784952 | -1.021537941 | 0.017581 | 0.2440599 |
| ARSF | 9.308734446 | -1.453536962 | 0.0045033 | 0.12158027 |
| VCX3A | 7.133346459 | 1.361766992 | 0.0030983 | 0.10090301 |
| FRMPD4 | 23.50665179 | -1.42688373 | 0.0112653 | 0.19979299 |
| BMX | 49.02586168 | -1.071946523 | 0.0004298 | 0.03061319 |
| MAGEB17 | 29.9664448 | -1.833018149 | 0.0021923 | 0.08351008 |
| RS1 | 5.811479335 | -1.357750781 | 0.0014268 | 0.06377923 |
| FTHL17 | 12.96919802 | -3.577250118 | 0.0036801 | 0.10905553 |
| SSX1 | 1.705356198 | 1.991156562 | 0.0014253 | 0.06377923 |
| GAGE2A | 10.00299595 | -3.496292615 | 0.0050659 | 0.12891356 |
| EZHIP | 15.92615456 | -2.208654343 | 0.0006784 | 0.03875618 |
| TENT5D | 2.620388845 | -1.523505738 | 0.0119688 | 0.20654283 |
| KLHL4 | 86.35706007 | 1.062433334 | 0.0111872 | 0.19916778 |
| TCEAL2 | 58.53247351 | 1.033805604 | 0.0130042 | 0.21367627 |
| TMSB15A | 121.1625034 | 1.724114789 | 5.90E-05 | 0.00798688 |
| NXF3 | 30.91891425 | -1.39986727 | 0.000394 | 0.02894798 |
| PLP1 | 14.86955441 | -2.068940327 | 1.43E-05 | 0.00330637 |
| H2BW1 | 15.84675573 | 1.128008909 | 0.0042089 | 0.11723586 |
| PAK3 | 68.1545684 | -1.435099035 | 0.0003037 | 0.02439081 |
| DCX | 4.775678958 | -1.682173583 | 0.0153438 | 0.23018426 |
| SLC6A14 | 1193.176589 | -1.06681594 | 0.005686 | 0.13752452 |
| ZCCHC12 | 17.66127735 | 1.387508405 | 5.09E-06 | 0.00157456 |
| CT55 | 3.095974697 | -2.640869849 | 0.0002663 | 0.02255956 |
| CT45A1 | 51.38141072 | -2.860337551 | 0.0054055 | 0.1336083 |
| SAGE1 | 115.85293 | -1.849957841 | 0.0295092 | 0.31198747 |
| GPR101 | 0.447412803 | 1.818728215 | 0.0103106 | 0.19310546 |
| SPANXB1 | 12.2779395 | 2.418192473 | 0.0263157 | 0.29640739 |
| MAGEC3 | 1.778576227 | 1.417629352 | 0.0043511 | 0.11940383 |
| SLITRK2 | 41.97361453 | -2.119391778 | 1.88E-05 | 0.0037196 |
| MAGEA9 | 36.06960451 | -2.329855252 | 0.0051059 | 0.12922092 |
| MAGEA8 | 8.619314663 | 1.313993015 | 0.0002857 | 0.02376329 |
| PASD1 | 6.027222592 | -2.361584683 | 0.0028289 | 0.0959407 |
| PNMA5 | 71.48674435 | -2.388497222 | 0.0001459 | 0.0157155 |
| PNMA6E | 3.227812843 | -2.105431976 | 0.0179472 | 0.24500133 |
| ATP2B3 | 6.677640646 | -1.954200846 | 1.07E-05 | 0.00279991 |
| TKTL1 | 16.38670767 | -2.089511215 | 9.66E-05 | 0.011794 |
| DEFB1 | 642.9484993 | -1.096774946 | 0.019402 | 0.25472493 |
| DEFB4A | 21.15467266 | 1.64132431 | 0.0026384 | 0.09358667 |
| USP17L2 | 0.535299967 | 1.225296822 | 0.0265809 | 0.29777845 |
| FGF20 | 5.161359146 | -1.453401864 | 0.0042684 | 0.11836012 |
| SLC7A2 | 1050.544739 | -1.155844005 | 0.0005526 | 0.03550231 |
| SFTPC | 12771.55423 | -2.234034571 | 0.0009771 | 0.04966147 |
| CHRNA2 | 2.708607635 | -1.324579054 | 0.0018557 | 0.07500051 |
| CHRNA6 | 16.30086567 | -1.230019753 | 0.003891 | 0.11245618 |
| EFCAB1 | 213.5523772 | -1.065509317 | 0.0031411 | 0.10116342 |
| ALKAL1 | 21.43257246 | -1.269388352 | 0.0047832 | 0.12604494 |
| OPRK1 | 103.0242691 | -1.61900309 | 0.0030619 | 0.10040828 |
| XKR4 | 28.04178651 | -2.860569881 | 1.22E-07 | 9.46E-05 |
| PENK | 507.1629767 | -2.698505169 | 1.91E-06 | 0.00072283 |
| CA8 | 138.2569484 | 1.132620146 | 0.0059913 | 0.1428598 |
| PPP1R42 | 7.355326932 | -1.515858155 | 0.0013925 | 0.06315972 |
| KCNB2 | 50.4051342 | -1.755700053 | 0.0019098 | 0.07668703 |
| PMP2 | 8.751934959 | -1.547710268 | 0.004313 | 0.11924136 |
| FABP4 | 208.225118 | -1.557729378 | 0.0004987 | 0.03376412 |
| DCAF4L2 | 30.9255716 | -5.344920132 | 0.0001056 | 0.01255839 |
| C8orf88 | 32.99146634 | -1.343264179 | 2.58E-05 | 0.00466234 |
| PKHD1L1 | 53.36968234 | -1.52920516 | 0.0001722 | 0.01736539 |
| PSCA | 372.4151261 | -1.734337098 | 6.55E-05 | 0.00868454 |
| SLURP1 | 19.6365902 | -1.229286735 | 0.0212734 | 0.26719249 |
| GPIHBP1 | 83.51113988 | -1.02668957 | 0.0075724 | 0.1642574 |
| FOXH1 | 26.2086406 | -1.270551344 | 0.0002359 | 0.02064453 |
| INSL6 | 1.104047625 | -1.216365971 | 0.021394 | 0.26780001 |
| CD274 | 1134.769131 | 1.072552637 | 0.0011481 | 0.05503256 |
| PTPRD | 978.3426699 | -1.239526077 | 0.0024455 | 0.0887814 |
| SH3GL2 | 24.18377842 | 2.240498157 | 1.36E-05 | 0.00328336 |
| IFNK | 0.720202224 | 1.649075609 | 0.0191095 | 0.25338019 |
| PRSS3 | 127.690357 | 1.239289371 | 0.009439 | 0.18412537 |
| DNAI1 | 37.01335131 | -1.022220444 | 0.0233422 | 0.2786496 |
| CNTFR | 133.5154713 | 1.072432832 | 0.0204472 | 0.26286679 |
| FOXD4L4 | 1.169688341 | -1.355659037 | 0.0050172 | 0.12866373 |
| RORB | 84.1574325 | -1.685609535 | 5.29E-05 | 0.00741449 |
| OGN | 95.77968538 | -1.536890498 | 0.0001134 | 0.01298195 |
| OMD | 105.3996105 | -1.054184254 | 0.0033398 | 0.10355178 |
| RNF183 | 80.60656397 | 1.015695607 | 0.0130035 | 0.21367627 |
| CRB2 | 25.05441149 | -1.390375329 | 0.0001444 | 0.0156611 |
| TTC16 | 45.44512267 | -1.111083885 | 0.0008068 | 0.04338492 |
| GFI1B | 5.651107929 | -1.760669413 | 0.0001134 | 0.01298195 |
| OBP2B | 2.084784288 | 1.441581681 | 0.0195598 | 0.25589135 |
| MYMK | 0.995830626 | -1.190032623 | 0.0476391 | 0.38524521 |
| PAEP | 37.21938244 | 1.814352065 | 5.19E-05 | 0.00735099 |
| CEND1 | 28.22785805 | 1.387480127 | 3.28E-05 | 0.00520779 |
| MUC6 | 40.31707813 | 1.506396551 | 0.0009135 | 0.04773521 |
| MUC2 | 62.38671999 | 2.112490975 | 0.0001783 | 0.01761315 |
| MUC5AC | 330.6076187 | -3.023297411 | 2.13E-07 | 0.00013639 |
| BRSK2 | 109.377369 | 1.109959641 | 0.0070937 | 0.15989801 |
| IGF2 | 2521.769221 | -1.032119335 | 0.0131678 | 0.2154103 |
| CNGA4 | 17.54634715 | -1.001180606 | 0.0043671 | 0.11966584 |
| CCKBR | 17.73180533 | -2.771608405 | 3.12E-05 | 0.00506954 |
| C11orf16 | 66.5674407 | -1.919609306 | 4.17E-05 | 0.0061427 |
| CALCA | 10.38584218 | -1.827387856 | 0.0006218 | 0.03697387 |
| MRGPRX3 | 24.47238613 | 1.424651116 | 0.0078984 | 0.16726327 |
| LRRC55 | 61.58450293 | -1.030345324 | 0.0133846 | 0.21704327 |
| GLYATL1 | 17.05806964 | 1.407259517 | 0.0017252 | 0.07151983 |
| MS4A6E | 2.530164685 | -1.127191991 | 0.0461435 | 0.38135812 |
| MS4A8 | 57.22659017 | -1.317868819 | 0.0297369 | 0.31333463 |
| MS4A15 | 71.84205 | -1.219549796 | 0.0049347 | 0.12803375 |
| SCGB2A1 | 15.5033255 | -1.171047181 | 0.028423 | 0.30726948 |
| SCGB1A1 | 2116.550006 | -1.726899671 | 0.0034596 | 0.10521112 |
| BATF2 | 431.5732691 | 1.017046671 | 0.0007265 | 0.04059332 |
| TSGA10IP | 5.033211745 | -1.20935628 | 0.0071199 | 0.15990967 |
| GAL3ST3 | 3.795922286 | -1.244861625 | 0.0350122 | 0.33987996 |
| SYT12 | 525.1466694 | 1.565709887 | 5.66E-05 | 0.00783021 |
| AP003419.1 | 0.526833961 | 1.710584853 | 0.0386198 | 0.35449557 |
| GAL | 268.9836082 | 1.152759286 | 0.006781 | 0.15587967 |
| TESMIN | 482.7256634 | 1.025381881 | 2.59E-05 | 0.00466234 |
| MYEOV | 602.8547637 | 1.191440811 | 0.0129354 | 0.21367627 |
| FGF19 | 169.1340946 | -1.677988316 | 0.0260272 | 0.29561886 |
| FGF4 | 2.009215601 | 2.404144756 | 0.0124448 | 0.21025744 |
| FGF3 | 1.165667443 | -2.123628369 | 0.0348767 | 0.33889988 |
| KRTAP5-7 | 3.411585231 | 1.195790504 | 0.0031155 | 0.10090301 |
| KRTAP5-8 | 4.415456356 | 1.306342245 | 0.0003674 | 0.02781992 |
| KRTAP5-9 | 30.25030933 | 1.469702715 | 0.0002924 | 0.02410997 |
| KRTAP5-10 | 9.582587473 | 1.15290305 | 0.0017272 | 0.07151983 |
| PHOX2A | 1.953608339 | -1.46901595 | 0.0495288 | 0.38877518 |
| MOGAT2 | 11.86428253 | -1.087029763 | 0.0333453 | 0.33293288 |
| THRSP | 1.784803276 | 1.504279363 | 0.0028234 | 0.0959407 |
| FAT3 | 121.0724152 | -1.568812492 | 0.0002143 | 0.0198969 |
| HEPHL1 | 356.4929918 | 1.154635865 | 0.0153489 | 0.23018426 |
| CNTN5 | 129.8093795 | 1.494209063 | 1.01E-05 | 0.00276957 |
| MMP7 | 3150.063739 | -1.24135148 | 0.0007846 | 0.04255705 |
| MMP20 | 6.873208822 | -1.160006232 | 0.0259832 | 0.29553309 |
| MMP27 | 4.544934903 | -2.956964812 | 3.06E-06 | 0.00109116 |
| MMP8 | 20.20780751 | -1.32283175 | 0.0005847 | 0.03562355 |
| MMP10 | 6987.923048 | -1.205512597 | 0.0208986 | 0.26465154 |
| MMP3 | 883.9840103 | 2.223080082 | 9.90E-07 | 0.00042692 |
| MMP13 | 3609.08463 | -1.900344265 | 0.0007845 | 0.04255705 |
| C11orf53 | 34.32648436 | -1.796058366 | 0.0006951 | 0.03910228 |
| PLET1 | 0.892287077 | 1.102441307 | 0.0206037 | 0.26329466 |
| HTR3B | 2.147272559 | -1.558418454 | 0.0399326 | 0.3586148 |
| HTR3A | 78.407284 | -1.528202984 | 0.0016936 | 0.07060308 |
| NXPE4 | 5.304334873 | -1.434933645 | 0.0030876 | 0.10084093 |
| APOA5 | 1.625570002 | 1.021537706 | 0.0492251 | 0.38877518 |
| FXYD2 | 27.06327466 | -1.291524756 | 0.0008327 | 0.04451764 |
| TREH | 7.167294561 | -1.103330808 | 0.001147 | 0.05503256 |
| ADAMTS8 | 85.56996114 | -1.037935883 | 0.007002 | 0.15900336 |
| CALML5 | 1718.263599 | -1.159288656 | 0.0466155 | 0.38258203 |
| OLAH | 5.487275579 | 1.807299017 | 5.85E-05 | 0.00798292 |
| ARMC3 | 63.01534301 | -1.231397282 | 0.0116503 | 0.20369956 |
| GJD4 | 2.428565516 | -1.202475209 | 0.0108776 | 0.19706137 |
| FXYD4 | 4.614209726 | -1.44291061 | 0.0035585 | 0.10645727 |
| MSMB | 200.6448982 | -1.637556364 | 0.0004403 | 0.03117615 |
| FRMPD2 | 15.79116158 | -1.212035708 | 0.0196213 | 0.25633387 |
| PCDH15 | 17.86822437 | -1.021145479 | 0.0072361 | 0.16134143 |
| MRLN | 30.79134329 | -1.437190688 | 0.0217284 | 0.26980141 |
| CDH23 | 424.666813 | -1.192146569 | 0.0001165 | 0.01315637 |
| SFTPA1 | 27657.27741 | -1.344740832 | 0.0227864 | 0.27593339 |
| C10orf99 | 504.1375149 | -1.256802142 | 0.0143757 | 0.22260809 |
| OPN4 | 2.052619353 | -1.168392069 | 0.0238124 | 0.28262595 |
| LIPN | 5.159807321 | -1.158096459 | 0.0033805 | 0.10417334 |
| LGI1 | 3.666765704 | -2.754027142 | 0.0020699 | 0.08100874 |
| HPSE2 | 49.59759486 | -1.720774528 | 5.32E-05 | 0.00741449 |
| WNT8B | 10.25449102 | 1.084432527 | 0.0008876 | 0.04664805 |
| PAX2 | 11.7706453 | -2.62226111 | 2.86E-05 | 0.00490987 |
| TLX1 | 128.9685339 | -2.679128267 | 1.48E-05 | 0.00335366 |
| CALHM1 | 4.477930255 | -1.008000018 | 0.0134724 | 0.21713788 |
| CALHM3 | 4.669500872 | 2.239281952 | 8.70E-07 | 0.00038937 |
| CFAP43 | 127.1215679 | -1.399289313 | 0.0001968 | 0.019067 |
| RBM20 | 81.40127321 | -1.302455652 | 3.08E-05 | 0.0050626 |
| TDRD1 | 4.799815206 | -1.196513414 | 0.0076926 | 0.1646576 |
| GFRA1 | 224.9292054 | -1.949022612 | 1.62E-06 | 0.00062691 |
| PNLIPRP3 | 30.38194375 | -1.895836878 | 0.0341263 | 0.33585014 |
| C10orf82 | 32.6407848 | -1.248797818 | 0.0282402 | 0.30600743 |
| FOXI2 | 14.38773368 | -1.690462684 | 0.0018515 | 0.07499231 |
| ADGRA1 | 7.587243358 | -2.064588071 | 0.0014041 | 0.06337552 |
| KCNA1 | 11.90291698 | -1.50115649 | 0.0080737 | 0.16866552 |
| MFAP5 | 478.3547759 | -1.104874433 | 0.0074021 | 0.16250565 |
| A2ML1 | 4710.685388 | -1.026614715 | 0.0344296 | 0.33740314 |
| PZP | 34.94053589 | -1.136372016 | 0.0045468 | 0.12224251 |
| KLRF2 | 3.728428317 | -1.561224533 | 0.0074631 | 0.16307222 |
| CLEC2A | 3.342234747 | -1.955045607 | 0.0175414 | 0.24401091 |
| TAS2R50 | 1.063186096 | -1.246684155 | 0.0072979 | 0.1617236 |
| PRB4 | 3.758826027 | -3.740547981 | 0.0005842 | 0.03562355 |
| GPRC5D | 208.2440288 | -1.208072042 | 0.0001551 | 0.01625562 |
| ART4 | 20.00433679 | -1.075440541 | 0.0039373 | 0.11289199 |
| PDE3A | 329.8446784 | -1.034883595 | 0.0003329 | 0.02594744 |
| SPX | 23.58744796 | -1.308390644 | 0.0014105 | 0.06339863 |
| SYT10 | 10.55922663 | 1.613967198 | 0.0085321 | 0.17374259 |
| MUC19 | 4.845622793 | -1.101122296 | 0.0387365 | 0.35503958 |
| PDZRN4 | 21.58311899 | -1.63532002 | 4.50E-05 | 0.00651924 |
| C1QL4 | 23.33659052 | -1.380994481 | 0.0002218 | 0.02007162 |
| AQP5 | 243.1614768 | -1.991790147 | 4.12E-05 | 0.0061427 |
| KRT75 | 504.843581 | -1.127792239 | 0.0195433 | 0.25587148 |
| KRT2 | 24.40096705 | -1.940921177 | 0.0002649 | 0.02253998 |
| KRT1 | 370.8195084 | -1.63582271 | 0.002659 | 0.09391915 |
| KRT77 | 141.2763175 | -1.864982331 | 0.0009969 | 0.05039232 |
| KRT76 | 16.2630776 | -1.864166516 | 0.0010155 | 0.05093083 |
| KRT4 | 2359.288488 | -2.54670513 | 7.21E-06 | 0.00208943 |
| KRT79 | 48.15223051 | -1.412307026 | 0.0006469 | 0.03797962 |
| KRT78 | 190.5046178 | -1.201648448 | 0.0038281 | 0.1116591 |
| PPP1R1A | 48.8947687 | 3.942279414 | 1.24E-21 | 2.31E-17 |
| INHBE | 66.21252587 | 1.190587752 | 5.00E-05 | 0.0071841 |
| WIF1 | 656.9475274 | -2.034947263 | 0.000541 | 0.03532869 |
| BEST3 | 11.71709316 | 1.943511943 | 0.0005645 | 0.03558143 |
| NTS | 20372.08308 | -1.844949617 | 0.0028178 | 0.0959407 |
| MGAT4C | 41.01690656 | -1.237603836 | 0.012252 | 0.2090955 |
| KERA | 7.880483952 | -1.009825886 | 0.0126864 | 0.21240612 |
| HAL | 93.22637544 | 1.398798525 | 0.0002823 | 0.02359073 |
| NR1H4 | 1.820129756 | -1.747599544 | 0.0086107 | 0.17438451 |
| SPIC | 5.105511442 | -2.579843284 | 0.0002218 | 0.02007162 |
| MYBPC1 | 112.170914 | -4.651322828 | 7.29E-11 | 1.93E-07 |
| ASCL1 | 37.16167647 | -1.228056919 | 0.01322 | 0.21569429 |
| ASCL4 | 15.57578233 | -2.479524942 | 9.42E-05 | 0.01162415 |
| FOXN4 | 19.24210265 | 2.064022347 | 0.0003807 | 0.02824815 |
| PLA2G1B | 26.54650577 | -1.119255723 | 0.0317281 | 0.3250067 |
| PIWIL1 | 10.26489707 | 2.298819639 | 0.0001831 | 0.01796978 |
| TUBA3C | 2.285512847 | 2.22057632 | 0.0003722 | 0.02783961 |
| AL359736.1 | 1.522362478 | -1.565580923 | 0.0016532 | 0.0695414 |
| TEX26 | 4.551352967 | -1.321841097 | 0.0145219 | 0.22356524 |
| MAB21L1 | 4.408802903 | -1.404865447 | 0.0043863 | 0.11970513 |
| SOHLH2 | 16.11068173 | -1.318090246 | 0.0017155 | 0.07135274 |
| FAM216B | 58.18680408 | -1.266248341 | 0.0194288 | 0.25489626 |
| CNMD | 12.86512888 | -1.66311897 | 0.0138661 | 0.21910486 |
| PCDH8 | 14.20835721 | -1.765821191 | 0.0086379 | 0.17474652 |
| OLFM4 | 171.1869114 | -1.178437761 | 0.0392385 | 0.35713162 |
| SLITRK5 | 101.496588 | -1.307893969 | 0.00388 | 0.11245618 |
| GPC5 | 19.91315511 | -1.090482726 | 0.0141476 | 0.22081015 |
| RNASE10 | 47.40422418 | 1.066108617 | 0.031683 | 0.3250067 |
| RNASE7 | 240.8137052 | 1.500036425 | 0.0005428 | 0.03532869 |
| JPH4 | 43.64086463 | -1.074673717 | 0.0005735 | 0.03558143 |
| RPL10L | 2.371891299 | 1.322798626 | 0.0054775 | 0.13423152 |
| PLEK2 | 1082.163636 | 1.229889604 | 3.71E-05 | 0.00573598 |
| VRTN | 2.541088048 | -1.316053769 | 0.0360755 | 0.34373451 |
| ESRRB | 28.94676035 | -1.258100417 | 0.0022527 | 0.08442309 |
| ZDHHC22 | 5.208033622 | -1.568027266 | 0.0080048 | 0.16817752 |
| SERPINA1 | 12093.24646 | 1.171948038 | 0.0014667 | 0.06478463 |
| SERPINA5 | 53.64760544 | 1.233495933 | 0.0012294 | 0.05788351 |
| GSC | 40.78344864 | 1.272521114 | 0.0054191 | 0.1336083 |
| TCL1B | 0.950132167 | -1.635995714 | 0.0372386 | 0.34784193 |
| TUNAR | 2.187504087 | -3.097810788 | 0.009 | 0.17900945 |
| DLK1 | 79.51739919 | -2.590040929 | 0.0028837 | 0.09691228 |
| GOLGA6L6 | 2.130218991 | -1.652593311 | 0.0430681 | 0.36971574 |
| GABRA5 | 86.59966215 | -1.968616639 | 0.0055007 | 0.13444582 |
| GOLGA6L7 | 58.7015076 | -1.313745763 | 0.0430008 | 0.36951751 |
| SCG5 | 182.4221161 | 1.399016276 | 1.11E-07 | 9.46E-05 |
| PHGR1 | 0.531892398 | -1.240638673 | 0.0421474 | 0.36699406 |
| DISP2 | 157.900491 | 1.389836954 | 1.44E-05 | 0.00330637 |
| FRMD5 | 111.2270165 | 1.20634566 | 0.0006465 | 0.03797962 |
| C15orf48 | 1317.281108 | 1.114031919 | 0.0008549 | 0.04505323 |
| SCG3 | 17.62117125 | -1.063302297 | 0.0061203 | 0.14444967 |
| NOX5 | 57.07464873 | 1.215080774 | 0.002517 | 0.09049164 |
| STRA6 | 1004.885251 | -1.200439865 | 0.0003072 | 0.02456155 |
| CCDC33 | 22.16025611 | -1.744844915 | 0.0011098 | 0.05417954 |
| ADAMTSL3 | 296.5089905 | -1.326313702 | 0.0001608 | 0.0166198 |
| MSLN | 1439.680364 | -1.370227125 | 0.0028916 | 0.09700317 |
| MSLNL | 22.9323473 | -1.935051745 | 7.36E-05 | 0.00948337 |
| SOX8 | 49.48408539 | -1.431121722 | 2.57E-05 | 0.00466234 |
| C1QTNF8 | 0.818828521 | -1.99355434 | 0.0169505 | 0.24034529 |
| HS3ST6 | 68.88577521 | -1.393183515 | 0.0034464 | 0.10498221 |
| NTN3 | 16.60889463 | -1.215058265 | 0.0001898 | 0.01852938 |
| PRSS27 | 289.6706731 | -1.137999862 | 0.0002233 | 0.02011015 |
| MTRNR2L4 | 4.284604759 | -1.073792921 | 0.000525 | 0.0347239 |
| TMEM114 | 5.621152799 | -1.2182442 | 0.021127 | 0.26625431 |
| GP2 | 5.808437457 | -2.571541739 | 0.0080386 | 0.16818462 |
| CRYM | 80.51547527 | -1.014378195 | 0.0076576 | 0.1646576 |
| VWA3A | 40.14083615 | -1.127038438 | 0.0081016 | 0.16905744 |
| HS3ST4 | 44.20153018 | -1.808986436 | 0.0077549 | 0.16535754 |
| NPIPB13 | 15.3929085 | -1.462558824 | 3.06E-05 | 0.0050626 |
| TRIM72 | 73.02045668 | -3.098857242 | 1.64E-10 | 3.79E-07 |
| PYDC1 | 10.69702518 | -1.318303523 | 0.011221 | 0.19957965 |
| IRX6 | 489.1034491 | -1.140824851 | 0.0010943 | 0.0535616 |
| SLC6A2 | 147.3092215 | 1.283891435 | 0.0080416 | 0.16818462 |
| MT2A | 9090.922015 | 1.535998094 | 1.87E-07 | 0.00012358 |
| MT1E | 1415.263312 | 1.201987021 | 0.0004448 | 0.03131824 |
| BEAN1 | 105.4296576 | 1.582690807 | 6.23E-07 | 0.00031226 |
| LRRC36 | 54.47662397 | -1.156670473 | 0.0003651 | 0.02775613 |
| CLEC18C | 1.992740196 | -1.142448653 | 0.0206869 | 0.26329466 |
| CNTNAP4 | 29.13099933 | 1.530026112 | 0.008323 | 0.17195248 |
| DYNLRB2 | 32.34157064 | -1.089749629 | 0.0059456 | 0.1421343 |
| JPH3 | 143.6680871 | 1.130658743 | 0.0219613 | 0.27160289 |
| DPEP1 | 79.20560505 | -1.175984114 | 0.0006426 | 0.03796284 |
| ALOX15 | 810.1138039 | -1.300167546 | 0.0137523 | 0.21789802 |
| SLC13A5 | 255.3683667 | -1.020536889 | 0.0350778 | 0.34032699 |
| TEKT1 | 46.53586131 | -1.019694589 | 0.0440797 | 0.37355957 |
| ALOX12 | 401.5005657 | -1.037143264 | 0.0015263 | 0.06630953 |
| RCVRN | 4.336994523 | -1.153487818 | 0.0012924 | 0.06023769 |
| MYH1 | 3.029178686 | -1.465778452 | 0.0185605 | 0.24896264 |
| DNAH9 | 105.5695159 | -1.070494049 | 0.0099991 | 0.19024956 |
| MYOCD | 36.05423302 | -1.738804798 | 3.89E-05 | 0.00595957 |
| LGALS9C | 41.59899984 | 1.19759676 | 0.004684 | 0.12449042 |
| SLC13A2 | 14.88865194 | -2.140074559 | 4.48E-06 | 0.00140847 |
| ASIC2 | 7.939809696 | -1.008237253 | 0.0076236 | 0.1642574 |
| UNC45B | 8.563401369 | -1.09823817 | 0.001039 | 0.05153474 |
| GAS2L2 | 29.35095914 | -1.2781055 | 0.0045003 | 0.12158027 |
| LHX1 | 24.28098348 | 2.37965945 | 0.0006539 | 0.03819087 |
| TBC1D3E | 2.50248042 | 1.864521916 | 0.0006956 | 0.03910228 |
| LRRC3C | 0.890032175 | -1.669458004 | 0.0032909 | 0.10330997 |
| CSF3 | 229.0210379 | -1.531680437 | 0.0017898 | 0.07345829 |
| KRT12 | 3.734153877 | -1.343717087 | 0.0058438 | 0.14042504 |
| KRT20 | 19.68024665 | -2.199137567 | 0.000127 | 0.01402761 |
| KRT23 | 995.2888298 | -2.466139811 | 1.41E-07 | 0.00010437 |
| KRT40 | 29.96185262 | -2.185769335 | 9.74E-05 | 0.01180584 |
| KRT31 | 391.7877443 | -1.47622322 | 0.004011 | 0.11352512 |
| KRT37 | 9.910640474 | -1.140204476 | 0.0208706 | 0.26465154 |
| KRT38 | 8.50978475 | -1.696370835 | 0.0039203 | 0.11275177 |
| KRT35 | 3.395893894 | -2.099202811 | 0.0033436 | 0.10355178 |
| KRT36 | 14.07446831 | -1.446309833 | 0.0002486 | 0.02145217 |
| KRT13 | 42316.36826 | -1.317715149 | 0.0134124 | 0.21707848 |
| CD300LG | 8.232324448 | -1.19838618 | 0.0110333 | 0.19852403 |
| CRHR1 | 13.42390579 | -1.621048414 | 0.0011192 | 0.05425033 |
| RPRML | 2.367287853 | 1.202964533 | 0.0109555 | 0.19788188 |
| HOXB8 | 89.70073447 | -1.170055138 | 0.0032756 | 0.10330997 |
| TTLL6 | 15.4198914 | -1.323454648 | 0.0197041 | 0.25686794 |
| GIP | 0.906474003 | 1.894348566 | 0.0021187 | 0.08239937 |
| CACNA1G | 36.81890476 | -1.152771255 | 0.0003175 | 0.02510852 |
| CA4 | 41.37636787 | -1.351220046 | 0.0032231 | 0.10250853 |
| MARCHF10 | 31.81783841 | -1.003252783 | 0.0028783 | 0.09690723 |
| KCNH6 | 7.086329719 | -1.11645159 | 0.0013532 | 0.06198332 |
| CACNG1 | 1.732120568 | -1.532233546 | 0.0064953 | 0.15042947 |
| BTBD17 | 4.538567311 | -2.478793492 | 0.0003934 | 0.02894798 |
| GPR142 | 8.493533305 | -1.920689242 | 0.001616 | 0.06875733 |
| GALR2 | 22.90281281 | -1.217138073 | 0.001276 | 0.05962693 |
| FOXJ1 | 512.4430862 | -1.286468862 | 0.0099891 | 0.19024956 |
| TMEM235 | 4.242669221 | -1.253050502 | 0.0182369 | 0.24675558 |
| CLUL1 | 63.80166284 | -1.313875426 | 0.0004545 | 0.03181644 |
| ADCYAP1 | 49.89311796 | -1.092647582 | 0.0069421 | 0.1584055 |
| AQP4 | 1067.186044 | -1.334286924 | 0.0107029 | 0.19526974 |
| CCDC178 | 6.330846509 | -1.117059727 | 0.0096235 | 0.18615749 |
| SYT4 | 1.733763352 | -1.894842392 | 0.0181561 | 0.24656915 |
| DCC | 331.8984191 | -1.312832306 | 0.0055245 | 0.13473169 |
| DYNAP | 6.847589404 | -2.073206256 | 0.0033401 | 0.10355178 |
| SERPINB12 | 107.2003455 | -2.72648083 | 2.29E-06 | 0.0008479 |
| SERPINB11 | 149.8199267 | -1.425718898 | 0.0072675 | 0.16171288 |
| GALR1 | 0.916248241 | -1.777069787 | 0.0294175 | 0.31166796 |
| TGM3 | 637.1653256 | -2.834917337 | 4.36E-07 | 0.00025259 |
| C20orf141 | 2.474338022 | 3.222400527 | 8.82E-07 | 0.00038937 |
| ADAM33 | 191.9885211 | -1.140345559 | 5.85E-05 | 0.00798292 |
| PCSK2 | 103.6791682 | -1.670407299 | 0.0025148 | 0.09049164 |
| INSM1 | 29.14151996 | -1.801462096 | 0.0009369 | 0.0485469 |
| NKX2-4 | 9.305949066 | 1.961246507 | 0.025234 | 0.29075521 |
| SSTR4 | 2.399402427 | -2.427477966 | 0.0026732 | 0.09397146 |
| CST2 | 44.29391898 | -1.054706813 | 0.0085762 | 0.17405423 |
| CST5 | 3.783042069 | -1.027571522 | 0.0471785 | 0.38445128 |
| GGTLC1 | 29.21691895 | -1.536792978 | 0.0084138 | 0.17276849 |
| VSX1 | 6.356679477 | 1.762979196 | 0.0005723 | 0.03558143 |
| DEFB124 | 3.292864684 | -1.225419387 | 0.0072876 | 0.16171288 |
| BPIFB2 | 19.96681649 | -2.363778236 | 0.0029097 | 0.09725138 |
| BPIFB1 | 2187.220831 | -1.366256779 | 0.028025 | 0.30454166 |
| LBP | 33.806626 | 1.104010793 | 0.0214756 | 0.26809856 |
| RIMS4 | 172.7030698 | 1.297187731 | 0.0192887 | 0.25431751 |
| PI3 | 12168.6498 | -1.168800932 | 0.0239265 | 0.28379864 |
| BCAS1 | 350.322414 | -1.090298318 | 0.0045316 | 0.12201037 |
| CYP24A1 | 1784.674802 | 2.261417623 | 5.76E-07 | 0.00029983 |
| CBLN4 | 17.5771478 | -1.574970494 | 0.0005654 | 0.03558143 |
| CTCFL | 66.30423702 | -1.811825207 | 0.0132658 | 0.21587135 |
| PCK1 | 8.916887282 | 1.994732744 | 0.0010062 | 0.05072115 |
| C20orf85 | 73.79061209 | -1.338300381 | 0.0423467 | 0.36743391 |
| SYCP2 | 377.0763861 | -1.227793309 | 6.06E-05 | 0.00814549 |
| CDH4 | 139.2400246 | 1.498119692 | 0.0005874 | 0.03562355 |
| NTSR1 | 19.63686084 | 2.148069082 | 6.72E-07 | 0.00032244 |
| EEF1A2 | 1444.837982 | 1.943994381 | 0.0002094 | 0.01961504 |
| ELANE | 4.9824517 | -1.637847398 | 0.0001785 | 0.01761315 |
| EFNA2 | 13.14193009 | -1.201435077 | 0.0136504 | 0.21748229 |
| ONECUT3 | 69.19682737 | -2.943764387 | 1.90E-08 | 1.95E-05 |
| CATSPERD | 6.439361617 | -1.004736508 | 0.0286236 | 0.30808558 |
| FUT6 | 126.6231874 | -1.094421894 | 0.0120437 | 0.2070641 |
| CD70 | 90.823859 | 1.612615485 | 1.03E-05 | 0.00276957 |
| FCER2 | 38.86455302 | -1.39732562 | 0.0015596 | 0.06744044 |
| EPHX3 | 615.4789184 | -1.05040643 | 0.0075499 | 0.1642574 |
| CYP4F8 | 3.167430961 | -1.769639521 | 0.0029925 | 0.09910563 |
| CRLF1 | 790.7144915 | 2.10451401 | 1.86E-09 | 2.88E-06 |
| ZNF208 | 26.0183348 | 1.237935716 | 0.0022903 | 0.08561364 |
| ZNF676 | 11.11550458 | 1.676929358 | 0.0021839 | 0.08351008 |
| ZNF98 | 5.74118383 | 1.686393042 | 0.006294 | 0.14668306 |
| ZNF536 | 15.03921635 | -2.783224119 | 2.47E-05 | 0.00457594 |
| TDRD12 | 63.34738034 | -2.05800563 | 4.16E-06 | 0.0013926 |
| FFAR1 | 1.640791375 | -1.011283733 | 0.0348558 | 0.33889388 |
| NPHS1 | 12.36736066 | -1.421598974 | 0.0007449 | 0.04088591 |
| KIRREL2 | 48.7297422 | -1.249192967 | 0.0030081 | 0.09929366 |
| CYP2A6 | 23.19472615 | -1.204602116 | 0.0009527 | 0.04895528 |
| CYP2A7 | 2.916182663 | -1.034219645 | 0.0220408 | 0.27204195 |
| CYP2F1 | 43.25543238 | -1.744282294 | 0.0018392 | 0.07482059 |
| CEACAM7 | 249.9443707 | -1.717628853 | 0.0051562 | 0.1299626 |
| PSG4 | 118.586698 | 1.340053569 | 0.0347539 | 0.33853014 |
| CD177 | 440.0449776 | -1.314659406 | 0.003761 | 0.11039547 |
| KCNN4 | 976.5692793 | 1.019946183 | 0.0027243 | 0.09517775 |
| IGFL3 | 57.69255928 | -1.175058744 | 0.042324 | 0.36740856 |
| HIF3A | 345.5800671 | -1.222379756 | 0.0015736 | 0.0678803 |
| ZNF541 | 31.64074553 | -1.032018228 | 0.004999 | 0.12866373 |
| CGB8 | 4.196707608 | 1.884720429 | 0.0130027 | 0.21367627 |
| IZUMO2 | 3.427690962 | 1.009923846 | 0.0486069 | 0.38776686 |
| SPIB | 287.9479256 | -2.060674745 | 5.26E-07 | 0.00028677 |
| LRRC4B | 108.7757264 | -1.229996666 | 8.09E-05 | 0.01023502 |
| KLK2 | 18.30897776 | -1.449103185 | 0.0075206 | 0.16413417 |
| KLK6 | 801.3494928 | 1.645593876 | 0.0032071 | 0.10250853 |
| ZNF534 | 3.73467428 | 1.105001215 | 0.006201 | 0.14557043 |
| ZNF578 | 15.84206187 | 1.81721958 | 1.15E-07 | 9.46E-05 |
| ERVV-1 | 11.47779946 | 3.66090782 | 5.85E-10 | 9.86E-07 |
| ERVV-2 | 5.46845431 | 3.012064641 | 0.0012287 | 0.05788351 |
| VSTM1 | 5.470386837 | 1.173670253 | 0.0071824 | 0.16062313 |
| KIR2DL1 | 3.429104192 | -1.096484768 | 0.0364161 | 0.3442763 |
| KIR3DL1 | 3.82107702 | -1.46757588 | 0.001603 | 0.06867764 |
| COX6B2 | 361.0731305 | -1.300488393 | 0.0021724 | 0.08343621 |
| AC020922.1 | 16.55253218 | -1.762705578 | 0.0007353 | 0.04084004 |
| NLRP11 | 21.55072057 | 1.453054898 | 0.0105355 | 0.19415441 |
| PEG3 | 72.24083221 | 1.074066595 | 0.0004869 | 0.03357978 |
| PCDH11Y | 1.231423195 | -2.186519477 | 0.0019952 | 0.07925769 |
| NLGN4Y | 251.3920648 | -1.280038234 | 0.0334416 | 0.33302547 |
| GGTLC3 | 0.982422272 | -1.431241926 | 0.0467234 | 0.38267844 |
| GGT2 | 22.3272197 | -1.304881525 | 0.0014746 | 0.06493521 |
| ZNF280A | 37.15013927 | -1.28989457 | 0.0043448 | 0.11940383 |
| IGLL1 | 19.17406994 | -1.643091121 | 0.0002283 | 0.0202633 |
| PIWIL3 | 1.200280811 | -1.842794483 | 0.0144879 | 0.22341245 |
| SEZ6L | 24.86120334 | -2.48842587 | 1.48E-06 | 0.00061002 |
| TMPRSS6 | 50.76777217 | 1.016351213 | 1.06E-05 | 0.00279991 |
| IL17REL | 42.96177876 | -2.103558374 | 3.12E-07 | 0.00018672 |
| MLC1 | 73.84114309 | -1.03944042 | 0.0029254 | 0.09743108 |
| CBSL | 29.76400247 | -1.197272595 | 0.0054188 | 0.1336083 |
| KRTAP19-1 | 59.92101432 | -2.844974488 | 4.46E-06 | 0.00140847 |
| C21orf62 | 4.181304908 | -1.032224697 | 0.0033529 | 0.10366588 |
| OLIG2 | 4.02863104 | -1.153783883 | 0.0311021 | 0.32215222 |
| B3GALT5 | 248.5159725 | -1.111733246 | 0.0080328 | 0.16818462 |
| TFF3 | 462.4258114 | -1.413716654 | 0.0006417 | 0.03796284 |
| TFF2 | 20.88609775 | -3.314173238 | 1.03E-05 | 0.00276957 |
| AIRE | 11.73607666 | -1.77050568 | 1.19E-05 | 0.00303662 |
| TSPEAR | 129.6822527 | -1.545126919 | 0.0004297 | 0.03061319 |
| FTCD | 48.84208205 | -1.312646418 | 0.0001086 | 0.01282998 |
