# Supplementary material for: A Combined RNA Signature Predicts Recurrence Risk of Stage I-IIIA Lung Squamous Cell Carcinoma
Source: Front Genet. 2021 Jun 14;12:676464. doi: 10.3389/fgene.2021.676464 (PMC8236863; doi:10.3389/fgene.2021.676464)
Supplement: Supplementary file 4 [file Table_4.DOCX]

**Supplementary Table 4.**

Correlation between lncRNA and recurrence in 161 patients with stage I-IIIA lung squamous cell carcinoma

| **Gene name** | **p** | **HR** | **95%CI** |
| --- | --- | --- | --- |
| AC015908.3 | 0.001343 | 4.157313 | 1.740166~9.931957 |
| DBET | 0.001384 | 4.399356 | 1.774478~10.907059 |
| LINC02418 | 0.001623 | 3.677507 | 1.636303~8.265009 |
| AC011676.1 | 0.002188 | 4.586865 | 1.73097~12.154646 |
| AC244517.5 | 0.002686 | 3.381415 | 1.52618~7.491885 |
| AC020637.1 | 0.002889 | 0.227394 | 0.085828~0.602461 |
| AL357552.2 | 0.005192 | 0.179842 | 0.053997~0.598977 |
| LINC02515 | 0.005406 | 3.080504 | 1.394294~6.805956 |
| AC079380.1 | 0.006417 | 2.960138 | 1.356448~6.459828 |
| BX640514.2 | 0.010482 | 2.867873 | 1.279917~6.425963 |
| HRAT92 | 0.010715 | 0.351396 | 0.157378~0.784602 |
| LINC02826 | 0.010749 | 0.209219 | 0.062887~0.696056 |
| LINC01778 | 0.011381 | 2.916373 | 1.273042~6.68103 |
| AC011468.3 | 0.013463 | 2.847448 | 1.241734~6.529543 |
| AC079336.3 | 0.013519 | 0.218964 | 0.065601~0.730863 |
| LINC02683 | 0.013962 | 2.650073 | 1.21841~5.763978 |
| FAM215A | 0.01477 | 0.323121 | 0.130295~0.801311 |
| AC093843.1 | 0.014933 | 0.342212 | 0.144307~0.811526 |
| AC125618.1 | 0.019042 | 0.368032 | 0.159589~0.848725 |
| AL133370.1 | 0.019207 | 0.3378 | 0.136183~0.837913 |
| AC022150.3 | 0.020662 | 2.502467 | 1.150704~5.442182 |
| AF212831.1 | 0.020726 | 0.374 | 0.162527~0.860629 |
| AL049820.1 | 0.021117 | 2.448251 | 1.143758~5.240561 |
| HORMAD2-AS1 | 0.023121 | 2.482307 | 1.132774~5.439613 |
| AC027117.2 | 0.023671 | 0.384084 | 0.167653~0.87992 |
| LINC01511 | 0.023856 | 0.364282 | 0.151707~0.874722 |
| LINC01322 | 0.024298 | 2.592113 | 1.131628~5.937509 |
| AC005544.1 | 0.024402 | 0.295137 | 0.101981~0.854143 |
| NCRNA00250 | 0.024502 | 0.325979 | 0.122736~0.865778 |
| AL031429.2 | 0.025783 | 0.29832 | 0.103007~0.86397 |
| LINC02066 | 0.026347 | 0.255191 | 0.076464~0.851675 |
| TLX1NB | 0.031441 | 0.343728 | 0.12993~0.909331 |
| AC091614.1 | 0.033658 | 2.292517 | 1.066228~4.92918 |
| AC109462.2 | 0.035816 | 0.412192 | 0.180176~0.94298 |
| AC105020.2 | 0.037427 | 0.399931 | 0.168706~0.948064 |
| AL391261.4 | 0.03819 | 2.262806 | 1.045458~4.897654 |
| AL035425.3 | 0.03892 | 0.437042 | 0.199222~0.958759 |
| PRRX2-AS1 | 0.040159 | 0.42056 | 0.183885~0.961855 |
| LINC00944 | 0.0416 | 2.271364 | 1.03171~5.000527 |
| AC022034.3 | 0.042108 | 0.387133 | 0.155031~0.966719 |
| AC040896.1 | 0.042951 | 2.194993 | 1.025257~4.6993 |
| PRKG2-AS1 | 0.044894 | 2.739895 | 1.023224~7.33664 |
| LINC01444 | 0.047346 | 2.713489 | 1.011758~7.277452 |
| AL023754.1 | 0.047466 | 0.451545 | 0.205713~0.991152 |
| AC022509.4 | 0.047983 | 2.178023 | 1.006933~4.71112 |
| AC148477.4 | 0.04908 | 2.21606 | 1.003212~4.895202 |
