# Supplementary material for: A Combined RNA Signature Predicts Recurrence Risk of Stage I-IIIA Lung Squamous Cell Carcinoma
Source: Front Genet. 2021 Jun 14;12:676464. doi: 10.3389/fgene.2021.676464 (PMC8236863; doi:10.3389/fgene.2021.676464)
Supplement: Supplementary file 5 [file Table_5.DOCX]

**Supplementary Table 5.**

Correlation between miRNA and recurrence in 161 patients with stage I-IIIA lung squamous cell carcinoma

| **Gene name** | **p** | **HR** | **95%CI** |
| --- | --- | --- | --- |
| hsa-mir-548x | 0.024449 | 2.541383 | 1.127756~5.726974 |
| hsa-mir-6825 | 0.013013 | 2.631329 | 1.226211~5.646573 |
| hsa-mir-1248 | 0.021298 | 0.402001 | 0.185078~0.873171 |
| hsa-mir-6814 | 0.042178 | 0.407073 | 0.171055~0.968739 |
