# Supplementary material for: A Combined RNA Signature Predicts Recurrence Risk of Stage I-IIIA Lung Squamous Cell Carcinoma
Source: Front Genet. 2021 Jun 14;12:676464. doi: 10.3389/fgene.2021.676464 (PMC8236863; doi:10.3389/fgene.2021.676464)
Supplement: Supplementary file 6 [file Table_6.DOCX]

**Supplementary Table 6.**

Correlation between mRNA and recurrence in 161 patients with stage I-IIIA lung squamous cell carcinoma

| **Gene name** | **p** | **HR** | **95%CI** |
| --- | --- | --- | --- |
| ERVV-1 | 0.012193 | 2.881928 | 1.259603~6.593753 |
| ERVV-2 | 0.004148 | 3.061625 | 1.424638~6.5796 |
| OR2T8 | 0.039807 | 2.506639 | 1.043746~6.019892 |
| TUBA3C | 0.049726 | 2.239305 | 1.000967~5.009643 |
| CDA | 0.006046 | 3.097042 | 1.381787~6.941498 |
| GIP | 0.039803 | 2.217244 | 1.037812~4.737053 |
| OLAH | 0.015815 | 2.686908 | 1.203969~5.996397 |
| TRIM58 | 0.043721 | 2.235581 | 1.022913~4.885871 |
| HMHB1 | 0.034181 | 2.287329 | 1.063654~4.918774 |
| TRIM36 | 0.027447 | 2.487215 | 1.106602~5.5903 |
| PCDHA10 | 0.011219 | 2.787292 | 1.2621~6.155613 |
| CSN3 | 0.015307 | 0.225099 | 0.067445~0.751277 |
| ODAM | 0.028409 | 0.380996 | 0.160743~0.903043 |
| KRTAP19-1 | 0.01215 | 0.344107 | 0.149483~0.792126 |
| SERPINB12 | 0.016546 | 0.362291 | 0.157928~0.831108 |
| IL17REL | 0.038136 | 0.401571 | 0.169512~0.951315 |
| UGT1A10 | 0.011477 | 0.328852 | 0.138837~0.778925 |
| UGT2B17 | 0.025949 | 0.390552 | 0.170733~0.893392 |
| HSPB3 | 0.011743 | 0.339524 | 0.146543~0.786641 |
| INSM1 | 0.020564 | 2.490476 | 1.150551~5.390868 |
| KCNB2 | 0.031805 | 0.403873 | 0.176509~0.92411 |
| AADACL2 | 0.041782 | 0.408497 | 0.17252~0.967248 |
| SMCP | 0.032107 | 0.415548 | 0.186136~0.927711 |
| LRRC3C | 0.027768 | 0.360837 | 0.145552~0.894545 |
| MYOG | 0.031134 | 0.308996 | 0.106204~0.899007 |
| SMIM32 | 0.029666 | 0.40936 | 0.183014~0.915644 |
| SMLR1 | 0.038909 | 0.40237 | 0.169586~0.954689 |
| TMEM212 | 0.023328 | 2.472579 | 1.130836~5.406308 |
| CRB2 | 0.006622 | 0.315083 | 0.136891~0.725226 |
| C20orf85 | 0.013727 | 2.66733 | 1.22237~5.820373 |
| ADGB | 0.021764 | 2.482234 | 1.141725~5.396645 |
| CHRNA2 | 0.013212 | 0.331798 | 0.138646~0.794032 |
| FTCD | 0.020316 | 0.37258 | 0.161828~0.8578 |
| FOXH1 | 0.034594 | 0.433651 | 0.199791~0.941253 |
| TAS2R50 | 0.005929 | 0.278146 | 0.1118~0.691998 |
| MSGN1 | 0.012352 | 0.363592 | 0.164591~0.803196 |
| ARMC3 | 0.04123 | 2.249873 | 1.032801~4.901166 |
| ECRG4 | 0.036482 | 2.296684 | 1.05369~5.005984 |
| C4orf51 | 0.048872 | 0.42017 | 0.177301~0.995722 |
| IGFL3 | 0.010779 | 0.300168 | 0.119021~0.757017 |
| RCVRN | 0.043988 | 0.419361 | 0.180028~0.976869 |
| CLEC18C | 0.041035 | 0.433129 | 0.194104~0.966497 |
| KCNH6 | 0.0251 | 0.353081 | 0.141987~0.878013 |
| MTRNR2L4 | 0.004741 | 0.270008 | 0.108831~0.669886 |
| SCG3 | 0.017927 | 2.550503 | 1.174743~5.537436 |
| PRSS12 | 0.022588 | 0.381727 | 0.166827~0.873454 |
| CYP2A7 | 0.026304 | 0.37632 | 0.158898~0.891245 |
| FAM71F1 | 0.031149 | 0.419928 | 0.190756~0.924422 |
