# Supplementary material for: A Combined RNA Signature Predicts Recurrence Risk of Stage I-IIIA Lung Squamous Cell Carcinoma
Source: Front Genet. 2021 Jun 14;12:676464. doi: 10.3389/fgene.2021.676464 (PMC8236863; doi:10.3389/fgene.2021.676464)
Supplement: Supplementary file 7 [file Table_7.DOCX]

**Supplementary Table7.**

Ninety-one prognostic genes significantly associated with RFS

| **Gene name** | **Coefficient** | **HR** | **95%CI** | **p-value** |
| --- | --- | --- | --- | --- |
| AC015908.3 | 1.425 | 4.157 | 1.74-9.932 | 0.001 |
| DBET | 1.481 | 4.399 | 1.774-10.907 | 0.000 |
| LINC02418 | 1.302 | 3.678 | 1.636-8.265 | 0.001 |
| AC011676.1 | 1.523 | 4.587 | 1.731-12.155 | 0.001 |
| AC244517.5 | 1.218 | 3.381 | 1.526-7.492 | 0.001 |
| AC020637.1 | -1.481 | 0.227 | 0.086-0.602 | 0.001 |
| AL357552.2 | -1.716 | 0.180 | 0.054-0.599 | 0.002 |
| LINC02515 | 1.125 | 3.081 | 1.394-6.806 | 0.003 |
| AC079380.1 | 1.085 | 2.960 | 1.356-6.46 | 0.004 |
| BX640514.2 | 1.054 | 2.868 | 1.28-6.426 | 0.007 |
| HRAT92 | -1.046 | 0.351 | 0.157-0.785 | 0.008 |
| LINC02826 | -1.564 | 0.209 | 0.063-0.696 | 0.005 |
| LINC01778 | 1.070 | 2.916 | 1.273-6.681 | 0.008 |
| AC011468.3 | 1.046 | 2.847 | 1.242-6.53 | 0.010 |
| AC079336.3 | -1.519 | 0.219 | 0.066-0.731 | 0.007 |
| LINC02683 | 0.975 | 2.650 | 1.218-5.764 | 0.011 |
| FAM215A | -1.130 | 0.323 | 0.13-0.801 | 0.010 |
| AC093843.1 | -1.072 | 0.342 | 0.144-0.812 | 0.011 |
| AC125618.1 | -1.000 | 0.368 | 0.16-0.849 | 0.015 |
| AL133370.1 | -1.085 | 0.338 | 0.136-0.838 | 0.014 |
| AC022150.3 | 0.917 | 2.502 | 1.151-5.442 | 0.017 |
| AF212831.1 | -0.984 | 0.374 | 0.163-0.861 | 0.016 |
| AL049820.1 | 0.895 | 2.448 | 1.144-5.241 | 0.017 |
| HORMAD2-AS1 | 0.909 | 2.482 | 1.133-5.44 | 0.019 |
| AC027117.2 | -0.957 | 0.384 | 0.168-0.88 | 0.019 |
| LINC01511 | -1.010 | 0.364 | 0.152-0.875 | 0.019 |
| LINC01322 | 0.952 | 2.592 | 1.132-5.938 | 0.019 |
| AC005544.1 | -1.220 | 0.295 | 0.102-0.854 | 0.017 |
| NCRNA00250 | -1.121 | 0.326 | 0.123-0.866 | 0.018 |
| AL031429.2 | -1.210 | 0.298 | 0.103-0.864 | 0.018 |
| LINC02066 | -1.366 | 0.255 | 0.076-0.852 | 0.017 |
| TLX1NB | -1.068 | 0.344 | 0.13-0.909 | 0.024 |
| AC091614.1 | 0.830 | 2.293 | 1.066-4.929 | 0.029 |
| AC109462.2 | -0.886 | 0.412 | 0.18-0.943 | 0.030 |
| AC105020.2 | -0.916 | 0.400 | 0.169-0.948 | 0.031 |
| AL391261.4 | 0.817 | 2.263 | 1.045-4.898 | 0.033 |
| AL035425.3 | -0.828 | 0.437 | 0.199-0.959 | 0.034 |
| PRRX2-AS1 | -0.866 | 0.421 | 0.184-0.962 | 0.034 |
| LINC00944 | 0.820 | 2.271 | 1.032-5.001 | 0.036 |
| AC022034.3 | -0.949 | 0.387 | 0.155-0.967 | 0.035 |
| AC040896.1 | 0.786 | 2.195 | 1.025-4.699 | 0.038 |
| PRKG2-AS1 | 1.008 | 2.740 | 1.023-7.337 | 0.037 |
| LINC01444 | 0.998 | 2.713 | 1.012-7.277 | 0.039 |
| AL023754.1 | -0.795 | 0.452 | 0.206-0.991 | 0.042 |
| AC022509.4 | 0.778 | 2.178 | 1.007-4.711 | 0.043 |
| AC148477.4 | 0.796 | 2.216 | 1.003-4.895 | 0.044 |
| ERVV-2 | 1.119 | 3.062 | 1.425-6.58 | 0.003 |
| MTRNR2L4 | -1.309 | 0.27 | 0.109-0.67 | 0.002 |
| TAS2R50 | -1.28 | 0.278 | 0.112-0.692 | 0.003 |
| CDA | 1.13 | 3.097 | 1.382-6.941 | 0.004 |
| CRB2 | -1.155 | 0.315 | 0.137-0.725 | 0.004 |
| IGFL3 | -1.203 | 0.3 | 0.119-0.757 | 0.007 |
| PCDHA10 | 1.025 | 2.787 | 1.262-6.156 | 0.008 |
| UGT1A10 | -1.112 | 0.329 | 0.139-0.779 | 0.008 |
| HSPB3 | -1.08 | 0.34 | 0.147-0.787 | 0.008 |
| KRTAP19-1 | -1.067 | 0.344 | 0.149-0.792 | 0.009 |
| ERVV-1 | 1.058 | 2.882 | 1.26-6.594 | 0.009 |
| MSGN1 | -1.012 | 0.364 | 0.165-0.803 | 0.009 |
| CHRNA2 | -1.103 | 0.332 | 0.139-0.794 | 0.009 |
| CSN3 | -1.491 | 0.225 | 0.067-0.751 | 0.008 |
| OLAH | 0.988 | 2.687 | 1.204-5.996 | 0.012 |
| SERPINB12 | -1.015 | 0.362 | 0.158-0.831 | 0.013 |
| FTCD | -0.987 | 0.373 | 0.162-0.858 | 0.016 |
| PRSS12 | -0.963 | 0.382 | 0.167-0.873 | 0.018 |
| KCNH6 | -1.041 | 0.353 | 0.142-0.878 | 0.019 |
| UGT2B17 | -0.94 | 0.391 | 0.171-0.893 | 0.021 |
| CYP2A7 | -0.977 | 0.376 | 0.159-0.891 | 0.021 |
| TRIM36 | 0.911 | 2.487 | 1.107-5.59 | 0.023 |
| LRRC3C | -1.019 | 0.361 | 0.146-0.895 | 0.022 |
| ODAM | -0.965 | 0.381 | 0.161-0.903 | 0.023 |
| SMIM32 | -0.893 | 0.409 | 0.183-0.916 | 0.025 |
| MYOG | -1.174 | 0.309 | 0.106-0.899 | 0.023 |
| FAM71F1 | -0.868 | 0.42 | 0.191-0.924 | 0.026 |
| KCNB2 | -0.907 | 0.404 | 0.177-0.924 | 0.026 |
| SMCP | -0.878 | 0.416 | 0.186-0.928 | 0.027 |
| HMHB1 | 0.827 | 2.287 | 1.064-4.919 | 0.03 |
| FOXH1 | -0.836 | 0.434 | 0.2-0.941 | 0.03 |
| IL17REL | -0.912 | 0.402 | 0.17-0.951 | 0.032 |
| SMLR1 | -0.91 | 0.402 | 0.17-0.955 | 0.033 |
| GIP | 0.796 | 2.217 | 1.038-4.737 | 0.035 |
| OR2T8 | 0.919 | 2.507 | 1.044-6.02 | 0.034 |
| CLEC18C | -0.837 | 0.433 | 0.194-0.966 | 0.036 |
| AADACL2 | -0.895 | 0.408 | 0.173-0.967 | 0.035 |
| TRIM58 | 0.805 | 2.236 | 1.023-4.886 | 0.039 |
| RCVRN | -0.869 | 0.419 | 0.18-0.977 | 0.038 |
| C4orf51 | -0.867 | 0.42 | 0.177-0.996 | 0.042 |
| TUBA3C | 0.806 | 2.239 | 1.001-5.01 | 0.044 |
| hsa-mir-6825 | 0.967 | 2.631 | 1.226-5.647 | 0.01 |
| hsa-mir-1248 | -0.911 | 0.402 | 0.185-0.873 | 0.017 |
| hsa-mir-548x | 0.933 | 2.541 | 1.128-5.727 | 0.02 |
| hsa-mir-6814 | -0.899 | 0.407 | 0.171-0.969 | 0.036 |

Abbreviations: gene: lncRNA, miRNA, mRNA ; HR, hazard ratio; CI,

confidence interval. RFS, recurrence-free survival
